# Genotypic variability and trait associations for cold stress tolerance in cultivated chickpea (*Cicer arietinum* L.) during the reproductive stage

**DOI:** 10.1371/journal.pone.0343120

**Published:** 2026-02-26

**Authors:** Deeksha Padhiar, Sarbjeet Kaur, Swarup K. Parida, Uday C. Jha, Kamal Dev Shama, Pagadala Venkat Vara Prasad, Kadambot H. M. Siddique, Harsh Nayyar

**Affiliations:** 1 Department of Botany, Panjab University, Chandigarh, India; 2 National Institute of Plant Genome Research, New Delhi, India; 3 Crop Improvement Division, Indian Institute of Pulses Research, Kanpur, India; 4 Department of Agricultural Biotechnology, Chaudhary Sarwan Kumar Himachal Pradesh Agricultural University, Palampur, India; 5 Department of Agronomy, Kansas State University, Manhattan, Kansas, United States of America; 6 The UWA Institute of Agriculture, The University of Western Australia, Perth, Australia; IRD: Institut de recherche pour le developpement, FRANCE

## Abstract

Chickpea (*Cicer arietinum* L.), a major winter legume in northern South Asia and Australia, frequently encounters low temperatures (0–15 °C) during reproduction, causing substantial yield losses. The present study involved screening two independent sets of 100 genotypes over consecutive winters to identify sources of reproductive-stage cold tolerance and to elucidate the underlying mechanisms. Following outdoor establishment, plants were exposed to controlled cold stress (15/7 °C day/night) during flowering and pod development (15 d) in walk-in growth chambers. Ten representative cold-tolerant (CT) and ten cold-sensitive (CS) genotypes were selected each year based on integrated performance across yield, physiological, biochemical, and reproductive traits for a detailed mechanistic analysis. Cold-sensitive genotypes exhibited severe dysfunction, characterized by high electrolyte leakage (50−59% above CT) and malondialdehyde (39−51% above CT), indicating membrane damage. Reduced chlorophyll content (21−23%), photosystem II efficiency (23−29%), and stomatal conductance (40−43%) impaired photosynthesis. Inadequate cryoprotectants (reduced by 25−58%) and antioxidants (reduced by 38−55%) caused oxidative damage. Reproductive collapse followed, with pollen viability and germination declining by 24−46%, stigma receptivity and ovule viability decreasing by 41−68%, and seed yields falling by 85−95%. Cold-tolerant genotypes-maintained homeostasis through integrated protection in terms of superior membrane stability, enhanced compatible solutes, and elevated antioxidant activities, which sustained photosynthesis and reproductive success, achieving better yields under cold stress. Principal component analysis revealed cold tolerance as an integrated system (PC1:72.6–81.3% variance), clearly separating the CT from the CS genotypes. Membrane stability, photosynthetic efficiency, and pollen viability emerged as diagnostic traits (r = 0.85–0.91 with yield, p < 0.001; heritability 70−99%). Tolerance operated independently of maturity (R² = 0.10–0.18), enabling donor identification across maturity classes. Twenty cold-tolerant genotypes were identified, spanning the early, medium, and late maturity groups, respectively. These findings establish a mechanistic understanding of reproductive-stage cold tolerance, provide vital selection markers, and identify genetic resources for breeding cold-resilient chickpea cultivars.

## 1. Introduction

Chickpea (*Cicer arietinum* L.) is a globally important legume crop valued for its high protein content, essential micronutrients, and biological nitrogen fixation [1,2]. In 2023, the global production of chickpea reached approximately 16.5 million tons, with India contributing nearly 75% [3]. Despite its importance in food security across South Asia, the Middle East, and the Mediterranean, chickpea production faces persistent challenges from abiotic stresses, particularly cold, heat, and drought, which limit the stability of the yield and restrict its expansion into climatically marginal areas.

Originating from the warm Mediterranean region, chickpea exhibits inherent sensitivity to low temperatures (<20/10 °C day/night), with critical thresholds between 4–6 °C triggering physiological dysfunction [4,5]. Depending on the sowing time and location, crops may encounter chilling stress during vegetative development or, more critically, during the reproductive phase when sensitivity is most pronounced [6,7]. Cold stress during reproduction is particularly harmful. Temperatures below 15 °C during flowering and early pod development disrupt microsporogenesis, impair pollen viability and germination, reduce stigma receptivity and ovule fertility, and trigger widespread flower and pod abortion, collectively resulting in yield losses exceeding 70% in susceptible genotypes [5,6,8]. This reproductive vulnerability represents a major constraint on chickpea productivity across South Asia and cool-season Mediterranean environments.

The physiological and biochemical basis of cold tolerance in chickpea is complex and multifaceted. At the cellular level, tolerance depends on maintaining membrane integrity and fluidity, as membrane destabilization disrupts compartmentation and metabolic function [9,10]. Photosynthetic stability, sustaining chlorophyll content, photosystem II efficiency, and stomatal conductance are essential for continued carbon assimilation and energy supply during stress [11]. Cold stress simultaneously induces osmotic and oxidative stress, requiring the coordinated accumulation of compatible solutes that provide osmotic adjustment, membrane stabilization, and contribute to reactive oxygen species (ROS) scavenging [12,13]. Activation of antioxidant defence systems, including enzymatic components and non-enzymatic antioxidants, mitigates oxidative damage reflected in reduced malondialdehyde (MDA) accumulation and lipid peroxidation [14,15]. These interconnected mechanisms ultimately determine whether reproductive processes can proceed normally, making them critical determinants of yield during cold stress [16].

Previous research has made important contributions to understanding cold tolerance in chickpeas. Early studies identified genetic variations in germplasm collections and wild relatives [17,18], while subsequent studies characterized vegetative-stage responses under controlled conditions [16,19]. Recent investigations have begun to elucidate reproductive-stage responses, demonstrating that cold-tolerant genotypes maintain reproductive organ function through enhanced antioxidant activity and cryoprotectant accumulation [6,20]. However, there are still significant knowledge gaps. Most previous studies have focused on the seedling or vegetative stages, examined small numbers of genotypes (typically <20), or evaluated limited trait sets without integrating physiological, biochemical, and reproductive responses into comprehensive tolerance profiles. Reproductive-stage cold tolerance, a critical determinant of yield stability, remains comparatively underexplored relative to its agronomic importance, and few studies have combined field and controlled environment evaluations to identify potential donor lines suitable for breeding deployment. Furthermore, the relative importance of different tolerance mechanisms, their hierarchical organization, and trait combinations that reliably predict field performance under reproductive-stage cold stress remain poorly understood.

This study addresses these critical gaps through a systematic multi-trait evaluation of cold tolerance during the reproductive phase. We screened two independent sets of 100 chickpea genotypes (200 unique genotypes in total) over two consecutive winter seasons, combining initial field evaluation with controlled cold stress imposed during flowering and early pod development in walk-in growth chambers. This experimental design enabled the assessment of yield performance under both optimal and cold stress conditions, while simultaneously measuring a comprehensive suite of physiological, biochemical, and reproductive traits under standardized stress conditions.

The specific objectives were as follows: (i) to identify cold-tolerant and cold-sensitive genotypes based on integrated mechanisms across two independent screening experiments; (ii) to characterize the physiological, biochemical, and reproductive trait profiles distinguishing tolerant from sensitive genotypes under reproductive-stage cold stress; (iii) to determine the traits and trait combinations most strongly associated with yield maintenance under cold stress using correlation and multivariate analyses; and (iv) to establish practical, trait-based selection criteria and identify donor genotypes spanning different maturity classes for breeding programs targeting cold-prone production environments.

## 2. Materials and methods

### 2.1. Experimental setup and growth conditions

Two independent sets of 100 chickpea genotypes **(S1 and S2 Tables in S1 File)** were obtained from the International Crops Research Institute for the Semi-Arid Tropics in Hyderabad, India. These two independent sets of 100 genotypes each year (totalling 200 unique genotypes) were screened for cold tolerance using multiple traits. Subsequently, a few contrasting genotypes (cold-tolerant and cold-sensitive) were identified from these two datasets based on their growth, physiological, biochemical, yield, and stability characteristics across two years. Each set, consisting of distinct genotypes, corresponded to a specific study year and was independently assessed over two consecutive years. Field experiments were conducted during the ‘rabi’ (winter) seasons of 2021–22 and 2022–23 at the Department of Botany, Panjab University, Chandigarh, India (30.75° N, 76.78° E). Sowing was carried out on November 1^st^ in both years, a period characterised by gradually declining night temperatures that typically impose cold stress during the reproductive stage. Prior to sowing, seeds were inoculated with *Mesorhizobium ciceri* (1.95 g kg ⁻ ¹ seed). Plants were grown in 4 kg pots containing a 3:1 sandy loam–to–sand mixture, supplemented with farmyard manure at a 3:1 soil-to-manure ratio.

Five seeds were initially sown per pot and subsequently thinned to three uniform seedlings after their emergence. The experiments were conducted using a randomized complete block design (RCBD) with three replicates. Each replicate comprised three pots per genotype and three plants per pot. Plants were maintained at the Department of Botany, Panjab University, Chandigarh, India, and assessed under field and controlled-environment conditions (S3 Table in S1 File). Irrigation was provided when needed. Regular temperature measurements (both maximum and minimum) and relative humidity were recorded from the sowing date to the flowering stage of the plants.

To evaluate the effects of cold stress, plants were initially raised under outdoor conditions with a light intensity of 1,300–1,500 µmol m ⁻ ² s ⁻ ¹ and 60–70% relative humidity, where average maximum temperatures gradually declined from 30–32 °C at sowing to approximately 15–18 °C, and minimum temperatures decreased from 14–16 °C to nearly 5–7 °C until the completion of the vegetative stage (S1a Fig in S1 File). The average temperature followed the same trend, indicating that the plants experienced a slight natural decrease in temperature without an abrupt cold exposure. At 78 days after sowing in the 1^st^ and 2^nd^ years, when all genotypes had initiated flowering, the plants were transferred to walk-in growth chambers for temperature treatments (days to flowering: S4 and S5 Tables in S1 File). The temperature regime in the growth chamber during the flowering-to-maturity phase followed a clearly defined sequence. During the acclimation phase, temperatures were gradually lowered (1 °C per day) from a 12 h day/12 h night regime of 25/15 °C to 15/7 °C, with a 12-h photoperiod (600 µmol m ⁻ ² s ⁻ ¹) and 65–70% relative humidity to impose controlled reproductive-stage cold stress. Cold stress (15/7 °C) was maintained for 15 d, whereas the control set of plants was grown at 25/15 °C for 15 d under similar light and RH conditions. Following stress, recovery was initiated by increasing the temperature by 2 °C per day until it reached 30/25 °C (day/night, 12-h light/12-h dark) in both the control and cold-stressed plants. The plants were then allowed to fully mature at this temperature. This contrast highlights that outdoor conditions imposed gradual cooling, whereas growth chamber treatments ensured precise cold stress at critical stages of plant development.

### 2.2. Trait measurement

Each year, 100 distinct genotypes were evaluated. Growth and yield traits were assessed at physiological maturity under both control and cold stress conditions, whereas physiological, biochemical, and reproductive traits were measured immediately after the 15-day cold stress treatment (on the 16^th^ day in the control and cold-stressed environments). The stress duration was kept constant for all genotypes by initiating cold exposure at the same reproductive stage and maintaining identical chamber conditions for each set.

In total, **31 traits** were measured and grouped into six categories:

1. **Growth traits:** plant height, biomass, harvest index
2. **Phenological traits** included days to flowering (DTF), days to podding (DTP), and days to maturity (DTM).
3. **Yield traits:** pod number, seed number, total seed weight per plant (seed yield), and 10-seed weight (seed size).
4. **Reproductive traits included** pollen viability, pollen germination, stigma receptivity, and ovule viability.
5. **Physiological traits included** membrane stability (electrolyte leakage), chlorophyll fluorescence (Fv/Fm), chlorophyll content (SPAD), relative leaf water content, stomatal conductance, and nodulation ability.
6. **Biochemical traits included** malondialdehyde (MDA), proline, trehalose, total soluble sugars, and antioxidant enzymes (superoxide dismutase (SOD), catalase (CAT), ascorbate peroxidase (APX), glutathione reductase (GR), ascorbic acid (AsA), and reduced glutathione (GSH).

### 2.3. Predicted yield (*Ŷ*_s_) and cold tolerance indexes

The relationship between seed yield under cold stress (Ys) and seed yield under control conditions (Yc) was analysed using linear regression for each year separately. The predicted seed yield under cold stress (Ŷs) was estimated from the regression equation derived between Yc and Ys. The slope (a) and intercept (b) of the regression were 0.94 and 2.35 in the first year, and 0.40 and 0.52 in the second year, respectively.

Cold-tolerance residuals (R) were calculated as the difference between observed and predicted seed yield under cold stress (R = Ys − Ŷs), following the framework described by [21]. These residuals represent yield variation under cold stress that is independent of yield potential under control conditions.

After calculating the residuals, they were used as dependent variables in regression analyses against multiple explanatory traits to identify parameters associated with cold tolerance or traits that are easier to assess.

(i) ratio of seed yield under cold to control conditions (Ys/Yc);
(ii) ratio of total biomass;
(iii) ratio of seed number per plant;
(iv) ratio of 10-seed weight (seed size);
(v) ratio of harvest index;
(vi) ratio of plant height;
(vii) days to flowering;
(viii) ratio of days to podding;
(ix) ratio of days to maturity; and
(x) ratio of physiological, biochemical, and reproductive traits.

Polynomial (Type II) regression models were applied where appropriate.

### 2.4. Physiological traits

Physiological traits were measured according to previously established protocols [6,22,23].

Membrane injury was quantified via electrolyte leakage (EL) by measuring the conductivity before and after heat treatment [22].

Lipid peroxidation was estimated as malondialdehyde (MDA) content using the thiobarbituric acid method, with absorbance measured at 532 nm, and the concentration calculated using an extinction coefficient of 155 mM ⁻ ¹ cm ⁻ ¹ [24].

The relative leaf water content (RLWC) was calculated from the fresh, turgid, and dry weights [25].

Stomatal conductance (gS) was measured using an SC-1 portable leaf porometer (Decagon Devices, Pullman, WA, USA) on leaves from the second or third node below the apex at 11:00 a.m. [23].

The SPAD chlorophyll index was estimated using a SPAD chlorophyll meter (Apogee Instruments, Logan, UT, USA) on marked leaves between 10:00 and 11:00 h [23].

Chlorophyll fluorescence (ChlF) was measured at 11:00 h using dark-adapted measurements with a modulated fluorometer (Model OS1-FL, Opti-Sciences, Tyngsboro, MA, USA) [23].

Carotenoids were extracted using 80% acetone and quantified spectrophotometrically at 440, 645, and 663 nm [26]. Nodulation ability was assessed by manual counting of nodules per plant [27].

### 2.5. Reproductive traits

Reproductive traits were assessed using previously established methods [6,20,22]. All reproductive samples were collected from plants grown under controlled chamber conditions one day before anthesis (stigma and ovule) or at anthesis (pollen).

Pollen viability was determined using 0.5% acetocarmine staining, with approximately 200 grains observed per sample, and viable grains were identified by their uniform size, shape, and strong red staining [22].

Pollen germination was assessed *in vitro* on nutrient medium (10% sucrose, 1,640 mM boric acid, 990 mM potassium nitrate, 812 mM magnesium sulfate, 1,269 mM calcium nitrate, pH 6.5), and the germination percentage was calculated as the proportion of grains with elongated pollen tubes [22,28].

Stigma receptivity was evaluated using an esterase-mediated chromogenic assay with α-naphthaleneacetic acid and fast blue B salt, scored on a 1–5 scale based on staining intensity [29].

Ovule viability was assessed using the triphenyl tetrazolium chloride (TTC) reduction assay, with viability scored on a 1–5 scale based on red pigmentation intensity [22].

### 2.6. Antioxidants

#### 2.6.1. Enzymatic antioxidants.

Antioxidant enzyme activity was determined using standard protocols [6,20,30–32].

Fresh tissue (~500 mg) was homogenized in ice-cold 50 mM phosphate buffer (pH 7.0–7.8) and centrifuged at 3,360–15,000 *g* for 5–15 min at 4 °C. The supernatants were used as enzyme extracts in all assays.

Superoxide dismutase (SOD) activity was assayed by monitoring the inhibition of nitro blue tetrazolium (NBT) photoreduction [33]. The reaction mixture contained enzyme extract, phosphate buffer (pH 7.8), methionine, NBT, EDTA, and riboflavin, and the absorbance was measured at 560 nm after 10 min of light exposure. The activity was expressed as units per mg of protein.

Catalase (CAT) activity was determined by monitoring the decline in absorbance at 410 nm due to H₂O₂ decomposition [30]. The activity was calculated using an extinction coefficient of 40 mM ⁻ ¹ cm ⁻ ¹ and expressed as mmol H₂O₂ decomposed per mg of protein.

Ascorbate peroxidase (APX) activity was measured by monitoring ascorbate oxidation at 290 nm [31]. Activity was calculated using an extinction coefficient of 2.8 mM ⁻ ¹ cm ⁻ ¹ and expressed as mmol of ascorbate oxidized per min per mg of protein.

Glutathione reductase (GR) activity was determined by following NADPH oxidation at 340 nm [32]. The activity was expressed as mmol GSSG reduced per min per mg of protein.

#### 2.6.2. Non-enzymatic antioxidants.

Ascorbate (AsA) content was determined following extraction in 6% TCA and reaction with 2% dinitrophenylhydrazine (DNPH) and thiourea, with absorbance measured at 530 nm after heating and sulfuric acid addition [34].

Reduced glutathione (GSH) was estimated using the DTNB method, with tissue homogenized in metaphosphoric acid and absorbance measured at 412 nm [35]. Both were quantified against standard curves and expressed as mg g ⁻ ¹ DW (AsA) or nmol g ⁻ ¹ DW (GSH).

### 2.7. Osmolytes

The osmolyte content was determined using standard colourimetric methods [6,20,36–39].

Proline was extracted using 3% sulfosalicylic acid, reacted with acidified ninhydrin, and the chromophore was extracted into toluene. The absorbance was measured at 520 nm, and the proline content was calculated using a standard curve [36].

Trehalose was extracted using 80% hot ethanol and quantified using the anthrone-TCA method [37,38].

Total soluble sugars were extracted in 80% ethanol through repeated extractions, reacted with anthrone reagent, and quantified at 625 nm using a glucose standard curve [39]. All osmolyte concentrations were expressed as nmol g ⁻ ¹ DW (proline and trehalose) or mg g ⁻ ¹ FW (total sugars).

### 2.8. Selection of Cold-tolerant and Cold-sensitive genotypes

A comprehensive multivariate analysis incorporating phenotypic, physiological, biochemical, and reproductive traits was performed for all 100 genotypes each year. This analysis consistently classified the genotypes into three groups: cold-tolerant (CT), moderately tolerant, and cold-sensitive (CS). The classification of 10 cold-tolerant (CT) and 10 cold-sensitive (CS) genotypes (separately each year) was based on their performance under cold stress. Genotypes that maintained higher yield traits, photosynthetic efficiency, membrane stability, relative water content, and chlorophyll content, along with improved pollen viability, germination, stigma receptivity, ovule viability, and elevated antioxidant and osmolyte accumulation, were designated as cold tolerant. In contrast, genotypes exhibiting sharp declines in these traits under cold stress were categorized as cold-sensitive. From these groups, ten representative CT and ten CS genotypes were selected from each dataset to serve as contrasting lines for a detailed statistical evaluation. This subset was used because reporting trait-by-trait data for all 100 genotypes would have been impractical and would not have improved the clarity of the results. All selections were derived from the same experimental dataset, and no separate experiments were performed. For each genotype, three samples per replicate were collected, averaged, and analysed [6].

### 2.9. Statistical analysis

Plants were grown using an RCBD. Each genotype was represented by three replicates per treatment, with each replicate consisting of one pot containing three plants (totalling nine plants per genotype per treatment). Data were analysed using two-way ANOVA (factors: genotype and treatment, **Table 1**) in RStudio with the *gvlma* package [40]. The Tukey test was calculated to assess the effects of genotype, treatment, and their interaction. Cluster analysis was performed using the *dendextend* package [41].

**Table 1:** Analysis of variance (ANOVA) and mean square values for phenological, growth, and yield traits of 200 chickpea genotypes under control and cold stress conditions.

Trait	Year	Genotype	Treatment	Replicate	G*T	Error
**Df**	–	99	1	2	99	398
***Phenological traits***						
**DTP**	1^st^ year	129***	22143***	128***	18	17
	2^nd^ year	74***	18282***	69*	8	23
**DTM**	1^st^ year	143***	75197***	179***	23	18
	2^nd^ year	132***	11189***	121**	19	21
***Growth traits***						
**PH**	1^st^ year	382.1***	2349.5***	5.1	15.8*	4.6
	2^nd^ year	303.4***	2030.3***	12.5	1.3	12.3
**Biomass**	1^st^ year	4***	4528***	0	2***	0
	2^nd^ year	6***	3286***	4***	2***	0
**HI**	1^st^ year	229**	40581***	381***	206***	33
	2^nd^ year	248***	36566***	718***	167***	25
***Yield traits***						
**FP**	1^st^ year	27***	24858 ***	1	12***	1
	2^nd^ year	61***	14308***	12***	6***	1
**10-sw**	1^st^ year	0.42***	124.4***	0.60***	0.43***	0.02
	2^nd^ year	0.55***	92.35***	0.01	0.51***	0.11
**TSW**	1^st^ year	1.0***	943.2***	0.5	0.7**	0.5
	2^nd^ year	1.2***	530.7***	0.3	0.4***	0.2
**SN**	1^st^ year	27 ***	22241***	12***	13***	1
	2^nd^ year	45***	13747***	13***	7***	1
***Physiological traits***						
**EL**	1^st^ year	48***	3597***	630***	1	1
	2^nd^ year	40***	3828***	965***	1***	1
**MDA**	1^st^ year	66***	3451***	1004***	2***	1
	2^nd^ year	46***	4752***	971***	2***	1
**RLWC**	1^st^ year	86***	7829***	558***	3**	2
	2^nd^ year	14***	14777***	1284***	6***	3
**SPAD**	1^st^ year	65***	26902***	365***	12	***1
	2^nd^ year	78***	5299***	810***	2**	1
**gS**	1^st^ year	985***	49713***	3117***	20	42
	2^nd^ year	811***	32501***	782***	3	5
**ChlF**	1^st^ year	0.015***	7.72***	0.16***	0.001	0.004
	2^nd^ year	0.016***	6.77***	0.10***	0.002*	0.001
**ND**	1^st^ year	46***	5588***	617***	2***	0
	2^nd^ year	43***	3698***	212***	0	1
***Reproductive traits***						
**PV**	1^st^ year	836***	26227***	1861***	17***	9
	2^nd^ year	627***	36017***	73**	23***	14
**PG**	1^st^ year	910***	26238***	3404***	17**	11
	2^nd^ year	604***	18950***	1425***	7*	5
**SR**	1^st^ year	5.11***	162.2***	32.41***	0.13	0.18
	2^nd^ year	5.6***	428.8***	2.1***	0.6***	0.2
**OV**	1^st^ year	5.65***	63.5***	31.1***	0.03	0.17
	2^nd^ year	7.52***	137.2***	2.13***	0.03	0.16
***Antioxidants***						
**SOD**	1^st^ year	29.9***	407.7***	273.5***	0.4	1.3
	2^nd^ year	20.6***	1001.3***	506.4***	0.9*	0.7
**CAT**	1^st^ year	2.41***	68.8***	27.3***	0.27***	0.14
	2^nd^ year	3.70***	60.4***	24.5***	0.25***	0.10
**APX**	1^st^ year	8.33***	55.0***	37.9***	0.16	0.13
	2^nd^ year	4.47***	71.6***	14.2***	0.34***	0.13
**GR**	1^st^ year	6.52***	67.2***	22.6***	0.12*	0.09
	2^nd^ year	7.81***	71.0***	33.8***	0.35***	0.08
**AsA**	1^st^ year	1574***	17918***	6422***	18***	7
	2^nd^ year	657***	17255***	5840***	27***	8
**GSH**	1^st^ year	226.2***	713.7***	950.3***	8.7***	2.1
	2^nd^ year	244***	3724***	2157***	17***	4
**Caro**	1^st^ year	1.08***	12.0***	28.7***	0.07*	0.05
	2^nd^ year	1.06***	17.4***	10.5***	0.03	0.08
***Osmolytes***						
**PRO**	1^st^ year	53.9***	1218.4***	1147.3***	2.2**	1.5
	2^nd^ year	51.5***	907.0***	1318.2***	2.6***	0.8
**TREH**	1^st^ year	2.44***	28.5***	56.9***	0.12***	0.03
	2^nd^ year	3.59***	28.7***	10.6***	0.16***	0.05
**TS**	1^st^ year	560***	6120***	1189***	20***	1
	2^nd^ year	162***	20184***	1260***	22***	3

**Abbreviations**: df: degree of freedom; DTP: Days to podding, DTM: days to maturity, PH: plant height, HI: harvest index, FP- filled pod; 10-SW: 10-seed weight; TSW-total seed weight; SN-seed number, EL, electrolyte leakage; MDA, malondialdehyde content; RLWC, relative leaf water content; SPAD, SPAD chlorophyll; gS, stomatal conductance; ChlF, chlorophyll fluorescence; NA, nodulation ability; SOD, superoxide dismutase; CAT, catalase; APX, ascorbate peroxidase; GR, glutathione reductase; AsA, Ascorbic acid; GSH, reduced glutathione; Caro, carotenoid; Pro, proline; TS, total sugars; Treh, trehalose; PV, pollen viability; PG, pollen germination; SR, stigma receptivity; OV, ovule viability ***p ≤ 0.001, **p ≤ 0.01, *p ≤ 0.05, ns = not significant.

Based on these results, a few representative contrasting genotypes were selected for detailed statistical evaluation from the same data set; no separate experiment was conducted. A nested ANOVA was used to analyse traits (Table 2). In this model, Category (cold-tolerant, CT; cold-sensitive, CS) was treated as a fixed factor, and Genotype nested within Category was also considered a fixed factor, as specific genotypes were of primary interest. Replication was included as a random factor to account for experimental variability. The final model structure was: Trait ~ Category + Genotype(Category) + Rep.

**Table 2:** Nested analysis of variance (ANOVA) and mean square values for physiological, biochemical, and reproductive traits of contrasting chickpea genotypes under cold stress.

Traits	1^st^ year				2^nd^ year			
	Category (C)	C*G (Genotype)	Residue	LSD (Genotypes within category)	Category (C)	C*G	Residue	LSD (Genotypes within category)
DF	1	18	38	–	1		38	–
*Phenological traits*								
DTF	128.9***	6.9	16.1	6.63	48.6	18.7	18.3	7.0
DTP	3153.7***	29.1*	13.7	6.13	806.6***	41.9***	0.06	5.24
DTM	1550.4***	55.3***	12.0	5.73	620.8***	94.1***	24.2	8.14
*Growth traits*								
PH	3735.1***	65.4***	6.8	4.30	820.6***	67.7***	3.01	2.86
Biomass	140.3***	1.50***	0.12	0.59	188.2***	1.78***	0.22	0.77
HI	5226.7***	161.0***	43.9	10.9	9275***	287***	36.3	9.96
*Physiological traits*								
EL	1224.0***	11.41***	1.31	1.26	1449.4***	14.80***	0.17	1.4
MDA	1784.7**	8.08***	1.44	1.26	1133.6***	6.17***	0.37	1.21
RLWC	1207.8***	55.2***	23.4	2.69	220.4***	13.04***	2.046	1.39
SPAD	669.2**	2.26***	2.26	1.38	256.6**	22.4***	0.93	1.68
gS	15105**	464.5***	30.8	6.73	19492***	114.4***	8.5	3.32
ChlF	0.44***	0.0017***	0.0001	0.021	0.6552***	0.006***	0.00038	0.025
NA	1785.4***	6.47***	0.64	1.05	503.4***	21.7***	0.61	1.41
*Reproductive traits*								
PV	13832**	86.8***	3.8	3.48	8882***	145.8***	1.7	3.62
PG	20832***	80.2***	5.8	3.35	5264***	114.6***	3.6	3.28
SR	72.60***	0.81***	0.45	0.13	150.42***	0.32***	0.19	0.264
OV	36.82 ***	0.78***	0.16	0.36	104.02***	0.63***	0.18	0.31
*Antioxidants*								
SOD	347.5***	12.4***	1.75	1.48	383.7 ***	5.72***	0.16	0.98
CAT	73.90 ***	1.64***	0.69	0.42	155.72***	0.43***	0.01	0.29
APX	230.46***	16.16***	0.42	0.333	193.00***	1.17***	0.031	0.37
GR	162.49***	20.02***	0.015	0.36	214.4***	4.03***	0.02	0.61
AsA	37660***	339***	4	5.94	21357***	186***	11.8	4.53
GSH	5980***	54.3***	0.5	2.39	6298***	109.1***	0.9	3.30
Caro	27.717**	0.29***	0.066	0.23	10.98***	0.91***	0.10	0.32
*Osmolytes*								
Pro	1678.0***	5.1***	2.03	1.51	1546.4***	28.1***	0.24	1.93
Treh	83.12***	0.39***	0.030	0.27	120.70***	1.76***	0.038	0.41
TS	16944 ***	59.0***	0.4	2.49	6429***	29.4***	1.5	2.15
*Yield traits*								
FP	1440***	1.87***	0.35	0.97	2065.0***	0.90***	0.15	0.64
10sw	8.78***	0.28***	0.01	0.21	17.3***	0.83***	0.06	0.40
TSW	29.17***	0.12***	0.03	0.30	29.3***	0.18***	0.02	0.28
SN	1456.9***	2.80***	0.38	1.02	2124***	1.31***	0.29	0.89

**Abbreviation**: df, degree of freedom; DTF: Days to flowering, DTP: Days to podding, DTM: days to maturity, PH: plant height, HI: harvest index, FP- filled pod; 10-SW: 10-seed weight; TSW-total seed weight; SN-seed number, EL, electrolyte leakage; MDA, malondialdehyde content; RLWC, relative leaf water content; SPAD, SPAD chlorophyll; gS, stomatal conductance; ChlF, chlorophyll fluoresence; NA, nodulation ability; SOD, superoxide dismutase; CAT, catalase; APX, ascorbate peroxidase; GR, glutathione reductase; AsA, Ascorbic acid; GSH, reduced glutathione; Caro, carotenoid; Pro, proline; TS, total sugars; Treh, trehalose; PV, pollen viability; PG, pollen germination; SR, stigma receptivity; OV, ovule viability ***p ≤ 0.001, **p ≤ 0.01, *p ≤ 0.05, ns = not significant.

Assumptions of ANOVA were evaluated before analysis. Normality of residuals was assessed using the Q–Q plots. Mean comparison was performed using LSD (p < 0.05). Each genotype was again represented by three pots (two plants per pot), with three replicates per treatment (nine plants per genotype). To examine trait relationships under cold stress, principal component analysis (PCA) was conducted using the *FactoMineR*, *factoextra*, and *ggplot2* packages [42–44]. For PCA analysis, all variables were z-standardised (mean-centred and scaled to unit variance) using the *scale. unit = TRUE* in the FactoMineR PCA function. This ensures that all traits contribute equally to the ordination regardless of their measurement units. Multivariate patterns across genotypes and traits were further visualised using heatmaps (*pheatmap* package; [45]), correlation plots (*corrplot* package; [46]), and radar plots (*fmsb* package; [47]). These analyses facilitated the identification of traits most strongly associated with cold tolerance.

## 3. Results

S1b Fig in S1 File depicts plants cultivated in both outdoor and growth chamber environments, emphasizing the impact of cold stress on their vegetative and reproductive parts. S1c Fig in S1 File illustrates the effects of cold stress on floral biology across genotypes that differ in cold tolerance.

### 3.1. Cold-tolerance residuals and yield-based+ regression

Over two years, 100 distinct genotypes were evaluated under both control and cold stress conditions to analyse the relationship between seed yield in optimal environments (Yc) and seed yield under cold stress (Ys). In both years, a moderate positive correlation was observed between seed yield under cold stress and control conditions (R² = 0.336 and 0.321, respectively; S2 Fig in S1 File), highlighting the significant genotypic variation in the yield response. Cold-tolerance residuals (Ys − Ŷs), derived from the regression of Ys on Yc, were strongly correlated with the seed-yield ratio (Ys/Yc), with R² values of 0.8 and 0.83 in the first and second years, respectively (S3 Fig in S1 File). These residuals represent variations in stress yield independent of yield potential and were therefore used as the primary indicators of cold tolerance in the subsequent analyses.

### 3.2. Potential traits explaining cold tolerance

#### 3.2.1. Phenology.

Phenological traits showed highly significant genotype × treatment interactions (P < 0.01), with greater variability under stress conditions (Table 3). Cold stress prolonged podding by 13–15 days and maturity by 10–15 days across both years.

**Table 3:** Ranges of key plant traits under control and cold stress across two study years.

Traits (units)	1^st^ year		2^nd^ year		Traits (Units)	1^st^ year		2^nd^ year	
	Range (control)	Range (Cold Stress)	Range (control)	Range (Cold Stress)		Range (control)	Range (Cold Stress)	Range (control)	Range (Cold Stress)
***Phenological traits***					***Reproductive traits***				
**DTF**	70-78		67-78		**PV (%)**	51.6-97.8	38.3-93	58.8-97.4	41.6-87.6
**DTP**	77-96	84-109	72-88	80-98	**PG (%)**	46.6-92.3	32.3-88.6	57.6-97.6	45.6-88.6
**DTM**	118-135	136-160	125-140	129-154	**SR (Scale 1–5)**	2-4.6	1-4	3-5	1-4.6
***Growth traits***					**OV (Scale 1–5)**	1.7-4.7	1-4.3	2-5	1-4.6
**PH (cm)**	59.7-77.3	56.2-76.1	37.4-69.1	33.4-65.3	***Antioxidants***				
**Biomass (g)**	5.8-10.1	0.07-5.06	4.0-10.0	0-5.9	**SOD (units/mg protein)**	10.2-25.1	11.5-27.1	14.8-22.6	15.5-25.3
**HI (%)**	30.5-50.6	0-41.4	28.3-46.7	0-44.1	**CAT (nmol H**_**2**_**O**_**2**_ **decomposed/min/mg protein)**	2.01-4.42	2.71-6.52	1.6-4.7	2.1-6.2
***Yield traits***					**APX (nmol oxidised donor/min/mg protein)**	2.25-6.80	2.68-8.47	2.7-6.4	3.2-8.5
**FP (Number/plant)**	10-20	0-12	5-23	0-13	**GR (nmol oxidised donor/min/mg protein)**	2.49-6.58	3.09-8.24	2.5-7.1	3.1-9.3
**10sw (g)**	1.05-1.96	0-1.79	1.5-3.9	0-2.2	**AsA (nmoles/g DW)**	90.7-159.6	100.1-177.9	78.1-129.8	87.4-153.8
**TSW (g)**	2.12-3.92	0-1.84	1.59-5.53	0-2.09	**GSH (nmoles/g DW)**	10.8-33.8	12.2-42.6	32.2-59.9	40.2-73.8
**SN (Number/plant)**	14-20	0-13	8-23	0-13	**Caro (mg/g DW)**	1.77-3.93	2.10-4.27	1.7-3.4	2.0-4.3
***Physiological traits***					***Osmolytes***				
**EL (%)**	12.9-23.8	15.2-28.8	11.3-23.4	14.5-28.4	**Pro (nmoles/g DW)**	11.1-23.0	13.6-29.8	12.5-25.4	14.5-32.4
**MDA (nmoles/g DW)**	16.1-31.2	17.7-36.2	18.1-29.5	20.5-35.5	**Treh (nmoles/g DW)**	1.80-4.02	2.13-5.31	1.04-3.95	1.37-5.48
**RLWC (%)**	76.1-92.2	69.8-87.7	88.3-96.9	78.3-87.8	**TS (mg/g DW)**	32.3-69.1	37.6-85.2	47.5-65.1	57.8-88.4
**SPAD (Units)**	27.6-45.9	14.1-32.5	15.3-36.2	9.0-29.9					
**gS (mmol/m** ^**2**^ **/s)**	44.2-113.4	28.1-98.4	47.4-102.6	32.4-87.6					
**ChlF (Fv/Fm)**	0.72-0.99	0.48-0.77	0.68-0.94	0.48-0.76					
**NA (number/plants)**	17.3-33.4	10.7-26.8	17.5-29.4	12.5-24.4					

**Abbreviation**: DTF: Days to flowering, DTP: Days to podding, DTM: days to maturity, PH: plant height, HI: harvest index, FP- filled pod; 10-SW: 10-seed weight; TSW-total seed weight; SN-seed number, EL, electrolyte leakage; MDA, malondialdehyde content; RLWC, relative leaf water content; SPAD, SPAD chlorophyll; gS, stomatal conductance; ChlF, chlorophyll fluorescence; NA, nodulation ability; SOD, superoxide dismutase; CAT, catalase; APX, ascorbate peroxidase; GR, glutathione reductase; AsA, Ascorbic acid; GSH, reduced glutathione; Caro, carotenoid; Pro, proline; TS, total sugars; Treh, trehalose; PV, pollen viability; PG, pollen germination; SR, stigma receptivity; OV, ovule viability

Regression of standardized yield residuals (Ys – Ŷs) against phenological traits revealed very weak associations; days to flowering (R² = 0.10–0.13), days to podding (R² = 0.14–0.18), and days to maturity (R² = 0.10–0.11) across both years (S4a-c, S5a-c Figs in S1 File). These negligible relationships indicate that phenology does not significantly contribute to cold tolerance variation in this germplasm panel, confirming that tolerance is independent of maturity-related traits. The heritability and genotypic variance are shown in Table 4.

**Table 4:** Summary statistics for key traits in chickpea genotypes under cold stress for both years.

Traits	Genotypic variance		Error		Heritability (%)	
	1^st^ year	2^nd^ year	1^st^ year	2^nd^ year	1^st^ year	2^nd^ year
*Phenological traits*						
DTF	0.09	0	14.0	17.3	0.64	0
DTP	27.5	9.33	18.8	14.7	59.3	38.7
DTM	24.7	21.3	17.2	26.3	58.8	44.7
*Growth traits*						
PH	64.5	48.2	7.2	12.3	89.8	79.5
Biomass	1.32	1.49	0.14	0.12	89.9	92.4
HI	117.4	114.2	37.6	22.6	75.7	83.4
*Yield traits*						
FP	10.5	11.3	0.24	0.17	97.7	98.4
10sw	0.26	0.27	0.021	0.06	92.4	79.9
TSW	0.22	0.17	0.013	0.013	94.3	93.0
SN	11.7	11.9	0.25	0.19	97.8	98.3
*Physiological traits*						
EL	9.64	8.60	0.94	0.18	91.0	97.9
MDA	13.8	9.59	0.68	0.98	95.2	90.6
RLWC	17.0	3.13	1.81	2.13	90.3	59.5
SPAD	14.6	12.7	1.27	1.27	91.9	90.9
gS	171.0	138.8	38.3	4.66	81.6	96.7
ChlF	0.003	0.003	0.0007	0.0001	80.3	95.6
NA	11.0	7.04	0.23	0.94	97.9	88.1
*Reproductive traits*						
PV	154.2	127.3	7.14	4.14	95.5	96.8
PG	176.3	108.4	8.6	3.5	95.3	96.7
SR	0.97	1.41	0.15	0.17	86.1	89.2
OV	0.95	1.28	0.16	0.17	85.2	88.0
*Antioxidants*						
SOD	4.14	4.24	1.19	0.34	77.6	92.5
CAT	0.62	0.88	0.10	0.07	85.5	92.3
APX	1.73	1.09	0.06	0.05	96.5	95.4
GR	1.32	1.78	0.04	0.03	96.5	97.9
AsA	306.2	147.7	6.27	6.95	97.9	95.5
GSH	50.2	54.3	0.81	2.32	98.4	95.8
Caro	0.23	0.19	0.04	0.06	83.9	73.9
*Osmolytes*						
Pro	12.2	11.1	0.82	0.43	93.6	96.2
Treh	0.57	0.80	0.01	0.04	97.0	94.8
TS	126.3	47.1	0.54	1.19	99.5	97.5

**Abbreviation**: DTF: Days to flowering, DTP: Days to podding, DTM: days to maturity, PH: plant height, HI: harvest index, FP- filled pod; 10-SW: 10-seed weight; TSW-total seed weight; SN-seed number, EL, electrolyte leakage; MDA, malondialdehyde content; RLWC, relative leaf water content; SPAD, SPAD chlorophyll; gS, stomatal conductance; ChlF, chlorophyll fluorescence; NA, nodulation ability; SOD, superoxide dismutase; CAT, catalase; APX, ascorbate peroxidase; GR, glutathione reductase; AsA, Ascorbic acid; GSH, reduced glutathione; Caro, carotenoid; Pro, proline; TS, total sugars; Treh, trehalose; PV, pollen viability; PG, pollen germination; SR, stigma receptivity; OV, ovule viability.

#### 3.2.2. Growth traits.

Plant height showed marginal reductions under cold stress (5.8–6.6% across years; Table 3), with very weak associations with cold tolerance residuals (R² = 0.07–0.22; S4d and S5d Figs in S1 File). indicating that height contributed little to the differences in tolerance.

Biomass accumulation declined sharply under cold conditions (70.9–72.1% reduction; Table 3), confirming strong growth inhibition. The biomass ratio showed a strong positive relationship with cold stress residuals (R² = 0.51 and 0.66; S4e and S5e Figs in S1 File), which demonstrated that genotypes maintaining biomass under cold stress exhibited higher cold tolerance. Heritability and genotypic variance are shown in Table 4.

#### 3.2.3. Yield traits.

Yield traits showed highly significant genotype × treatment interactions (P < 0.01), with cold stress causing consistent reductions in filled pods (76.5–76.9%), 10-seed weight (43.8–50.3%), total seed weight (82.1–83.2%), and seed number (74.0–75.3%) relative to controls across both years (Tables 3, 4).

Regression analysis revealed strong positive associations between the yield component ratios and cold tolerance residuals. The filled pod ratio, seed number ratio, and 10-seed weight ratio were strongly associated with cold tolerance residuals in Year 1 (R² = 0.51–0.59; S6 Fig in S1 File). and Year 2 (R² = 0.44–0.66; S7 Fig in S1 File), indicating that genotypes sustaining reproductive success and seed size under cold stress demonstrated greater cold tolerance.

#### 3.2.4. Physiological traits.

Leaf injury characteristics exhibited significant genotype × treatment interactions (P < 0.01). Cold stress increased electrolyte leakage (EL) by 25.6–26.2% and malondialdehyde (MDA) by 18.2–23.7% across both years, while significantly decreasing relative leaf water content (8.7–11.1%), SPAD chlorophyll (24.9–37.1%), stomatal conductance (20.5–22.0%), chlorophyll fluorescence (26.1–27.0%), and nodulation ability (23.9–31.3%), highlighting substantial genotypic variability in cold stress responses. Physiological traits are summarized as ranges across genotypes under control and cold stress conditions (Table 3), with corresponding genotypic variance, error variance, and heritability estimates (Table 4).

#### 3.2.5. Reproductive traits.

Reproductive function shifted toward lower ranges under cold stress across both years (Tables 3, 4). Cold stress reduced pollen viability by 17.9–19.2%, pollen germination by 14.9–19.9%, stigma receptivity by 32.7–43.8%, and ovule viability by 21.7–30.9%, indicating marked impairment of reproductive competence despite substantial genotypic variability.

#### 3.2.6. Antioxidants.

Antioxidant enzyme activity significantly increased under cold stress. Superoxide dismutase increased by 9.2–13.3%, catalase by 23.4–32.8%, ascorbate peroxidase by 15.3–16.3%, and glutathione reductase by 17.5–17.9% across both years (Tables 3, 4). Non-enzymatic antioxidants also increased; ascorbate by 9.85–10.6%, glutathione by 11.2–12.0%, and carotenoids by 14.3–14.7% (Tables 3, 4), although the extent varied between genotypes. To account for genotypic variability, antioxidant responses are presented as ranges across genotypes under both control and cold stress conditions (Tables 3 and 4).

#### 3.2.7. Osmolytes.

Osmolyte accumulation increased under cold stress in both years (Tables 3 and 4). Proline increased by 13.4–18.4%, trehalose by 18.8–19.1%, and total sugars by 14.5–21.5%, indicating improved osmotic adjustment across the germplasm panel despite significant inter-genotypic variability.

Each physiological, biochemical, and reproductive trait ratio was independently regressed against residual grain yield for both experimental years (S6 Table in S1 File). The analyses revealed moderate yet statistically significant associations, with reproductive trait ratios showing comparatively stronger relationships, whereas physiological and biochemical traits exhibited moderate associations. These results indicate that yield variation under cold stress reflects the combined influence of multiple trait categories rather than dependence on any single trait.

### 3.3. Cluster analysis

Cluster analysis was conducted using standardised (Z-score transformed) physiological, reproductive, biochemical, and yield traits measured under cold stress conditions. Traits included in the clustering were pre-selected based on regression analysis, showing moderate to high and statistically significant associations with residual seed yield. In contrast, phenological traits exhibited weak relationships with residual yield and were therefore excluded from the clustering analysis, as they contributed minimally to explaining yield variation under cold stress. This hierarchical clustering grouped genotypes based on multivariate similarity, resulting in distinct clusters representing differential cold stress responses**.**

**Fig 1:**
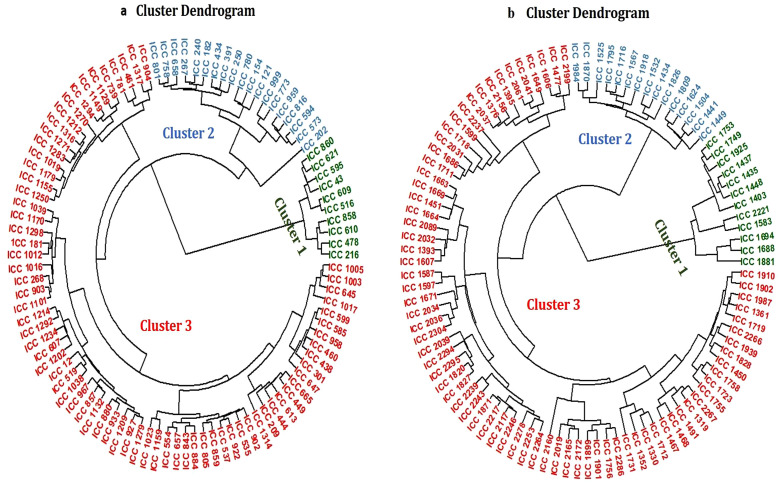
a: Cluster analysis of genotypes evaluated in the first year. Cluster 1 represents tolerant genotypes, Cluster 2 moderately tolerant genotypes, and Cluster 3 sensitive genotypes. b: Cluster analysis of genotypes evaluated in the second year. Cluster 1 represents tolerant genotypes, Cluster 2 moderately tolerant genotypes, and Cluster 3 sensitive genotypes.

In the first year (Fig 1a), Cluster 1 comprised tolerant genotypes (e.g., ICC 43, ICC 216, ICC 610, ICC 860, ICC 621, ICC 595, ICC 516, ICC 858, ICC 478, ICC 609), characterized by earlier phenological development, higher antioxidant activity, greater accumulation of osmolytes, and higher yields. Cluster 2 included moderately tolerant genotypes, while Cluster 3 represented sensitive genotypes (e.g., ICC 1212, ICC 1155, ICC 1250) showing delayed phenological development, low antioxidant activity, poor osmolyte accumulation, and low yields.

In the second year (Fig 1b), Cluster 1 consisted of tolerant genotypes (e.g., ICC 1881, ICC 1753, ICC 1583, ICC 1435, ICC 2221, ICC 1448, ICC 1749, ICC 1925, ICC 1403) characterized by earlier phenological development, higher antioxidant activity, greater osmolyte accumulation, and higher yields. Cluster 2 represented moderately tolerant genotypes, while Cluster 3 comprised highly sensitive genotypes (e.g., ICC 1901, ICC 1477, ICC 1395, ICC 1376, ICC 1731, ICC 1718, ICC 1451) exhibiting delayed phenological traits, low antioxidant activity, poor osmolyte accumulation, reduced reproductive success, and low yields.

### 3.4. Selection of contrasting genotypes for detailed analysis

Comprehensive datasets were obtained for all 100 genotypes across both years. For detailed statistical interpretation, ten cold-tolerant (CT) and ten cold-sensitive (CS) genotypes representing the most contrasting phenotypes were selected from each year’s dataset based on their integrated multi-trait performance under cold stress for in-depth characterisation. In the first year, CT genotypes (ICC 43, ICC 216, ICC 478, ICC 516, ICC 595, ICC 609, ICC 610, ICC 621, ICC 858, ICC 860) were selected from the highest tolerant cluster (Cluster 1). In contrast, Cluster 3 comprised a larger set of cold-sensitive genotypes, from which ten genotypes (ICC 268, ICC 535, ICC 599, ICC 859, ICC 922, ICC 967, ICC 1101, ICC 1129, ICC 1212, ICC 1314) were strategically selected based on exhibiting the highest percentage change across yield, physiological, biochemical, and reproductive traits under cold stress relative to control conditions.

In the second year, CT genotypes (ICC 1403, ICC 1435, ICC 1437, ICC 1448, ICC 1583, ICC 1749, ICC 1753, ICC 1881, ICC 1925, ICC 2221) were selected from the highest tolerant cluster. Conversely, although Cluster 3 contained a broader group of cold-sensitive genotypes, ten lines (ICC 1395, ICC 1376, ICC 1451, ICC 1477, ICC 1606, ICC 1607, ICC 1649, ICC 1756, ICC 1899, ICC 1901) displaying the highest percentage changes in yield, physiological, biochemical, and reproductive traits under cold stress relative to controls were chosen for detailed analysis.

### 3.5. Detailed evaluation of selected cold-tolerant and cold-sensitive genotypes

#### 3.5.1. Phenological traits.

**Fig 2:**
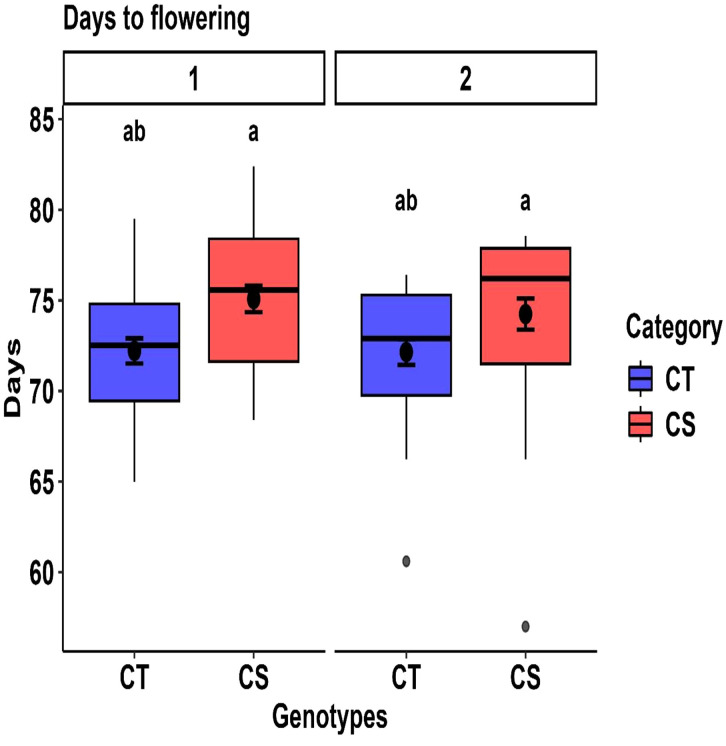
Year-wise comparison of days to flowering (DTF) between cold-tolerant (CT) and cold-sensitive (CS) genotypes. Boxplots illustrate the distribution of DTF within each tolerance group for Years 1 and 2. Different letters above the boxes indicate statistically significant differences between CT and CS groups within the same year, based on Tukey’s HSD test (P < 0.05).

The mean days to flowering for cold-tolerant genotypes was 72 days compared to 74–76 days for cold-sensitive genotypes across both years (Fig 2). While CT genotypes flowered slightly earlier, these differences were not statistically significant, suggesting that the flowering time remained consistent between the groups. The phenological traits of the contrasting genotypes, including percentage changes, are summarized in Table 5.

**Table 5:** Comparative analysis of yield and physiological, oxidative, and biochemical traits in chickpea; percentage decrease in cold-sensitive genotypes calculated relative to the mean of cold-tolerant genotypes under cold stress. (Trait units are as defined in Table 3).

Traits	1^st^ year			2^nd^ year		
	Cold-tolerant	Cold-sensitive	Percent change	Cold-tolerant	Cold-sensitive	Percent change
*Phenological traits*						
DTF	72 ± 0.88	75 ± 1.42	5	72 ± 1.26	74 ± 0.53	3
DTP	86 ± 0.13	100 ± 1.70	17	86 ± 0.27	93 ± 0.69	8
DTM	140 ± 0.33	150 ± 0.40	7	138 ± 0.78	148 ± 0.88	4
*Growth traits*						
PH	72.1 ± 0.16	56.4 ± 0.75	21.8	57.3 ± 0.22	49.8 ± 0.25	12.9
Biomass	4.08 ± 0.12	1.02 ± 0.02	74.8	4.19 ± 0.07	0.64 ± 0.01	84.5
HI	37.7 ± 1.72	19.1 ± 0.92	49.3	37.3 ± 3.25	12.4 ± 0.40	66.7
*Physiological traits*						
EL	17.7 ± 0.92	26.8 ± 0.82	–50	16.7 ± 1.09	26.5 ± 1.18	–58.8
MDA	21.3 ± 1.01	32.2 ± 1.23	–51.1	22.3 ± 1.22	31.0 ± 1.31	–39
RLWC	84.3 ± 0.25	75.0 ± 1.01	10.9	86.1 ± 0.83	82.3 ± 1.22	4.5
SPAD	28.6 ± 0.28	21.9 ± 0.83	23.4	19.5 ± 1.29	18.7 ± 1.32	21.5
gS	79.1 ± 1.92	47.4 ± 2.82	40.1	84.4 ± 1.79	77.7 ± 1.04	42.6
ChlF	0.8 ± 0.02	0.6 ± 0.02	22.9	0.7 ± 0.002	0.5 ± 0.010	28.6
NA	23.5 ± 1.06	12.6 ± 0.78	46.2	21.8 ± 0.72	16.1 ± 0.41	26.5%
*Reproductive traits*						
PV	85.7 ± 1.65	55.3 ± 2.66	35.4	81.6 ± 1.09	57.3 ± 1.31	29.8
PG	79.9 ± 1.70	43.1 ± 2.31	46.0	78.2 ± 1.05	59.4 ± 1.22	24
SR	3.8 ± 0.25	1.6 ± 0.2	57.92	4.2 ± 0.24	1.4 ± 0.033	67.6
OV	3.8 ± 0.32	2.2 ± 0.21	41.14	4.1 ± 0.25	1.8 ± 1.77	56.8
*Antioxidants*						
SOD	19.1 ± 0.25	24.3 ± 0.53	–27.41	17 ± 0.89	22.0 ± 0.92	–29.8
CAT	5.8 ± 0.29	3.6 ± 0.28	38.23	5.9 ± 0.29	2.6 ± 0.28	55
APX	7.9 ± 0.25	4.0 ± 0.18	49.70	7.8 ± 0.25	4.0 ± 0.28	48.4
GR	7.3 ± 0.28	4.0 ± 0.24	45.37	7.8 ± 0.21	4.2 ± 0.24	45.8
AsA	164.5 ± 3.53	114.4 ± 3.4	30.4	139.6 ± 1.99	1.5.2 ± 2.74	24.6
GSH	38.1 ± 1.38	18.1 ± 1.29	52.4	64.1 ± 1.91	43.6 ± 1.64	32
Caro	3.7 ± 0.19	2.4 ± 0.20	36.4	3.5 ± 0.20	2.6 ± 0.12	24.6
*Osmolytes*						
TS	81.0 ± 1.39	47.3 ± 1.62	41.5	84.4 ± 2.16	63.7 ± 1.93	24.5
Pro	28.0 ± 1.80	17.4 ± 150	37.8	27.7 ± 1.62	17.5 ± 1.50	36.7
Treh	4.8 ± 0.28	2.4 ± 0.24	49.1	4.9 ± 0.19	2.1 ± 0.16	57.9
*Yield traits*						
FP	11.2 ± 0.5	1.3 ± 0.04	88.1	12 ± 0.16	1.66 ± 0.05	95.7
10-SW	1.59 ± 0.012	0.63 ± 0.010	59.7	1.63 ± 0.01	0.55 ± 0.03	65.9
TSW	1.53 ± 0.12	0.13 ± 0.002	86.2	1.46 ± 0.13	0.20 ± 0.01	95.4
SN	11.5 ± 0.48	1.7 ± 0.05	86.0	12.5 ± 0.24	1.68 ± 0.04	95.2

**Abbreviations**: DTF: Days to flowering, DTP: Days to podding, DTM: days to maturity, PH: plant height, HI: harvest index, FP- filled pod; 10-SW: 10-seed weight; TSW-total seed weight; SN-seed number, EL, electrolyte leakage; MDA, malondialdehyde content; RLWC, relative leaf water content; SPAD, SPAD chlorophyll; gS, stomatal conductance; ChlF, chlorophyll fluorescence; NA, nodulation ability; SOD, superoxide dismutase; CAT, catalase; APX, ascorbate peroxidase; GR, glutathione reductase; AsA, Ascorbic acid; GSH, reduced glutathione; Caro, carotenoid; Pro, proline; TS, total sugars; Treh, trehalose; PV, pollen viability; PG, pollen germination; SR, stigma receptivity; OV, ovule viability.

**Fig 3:**
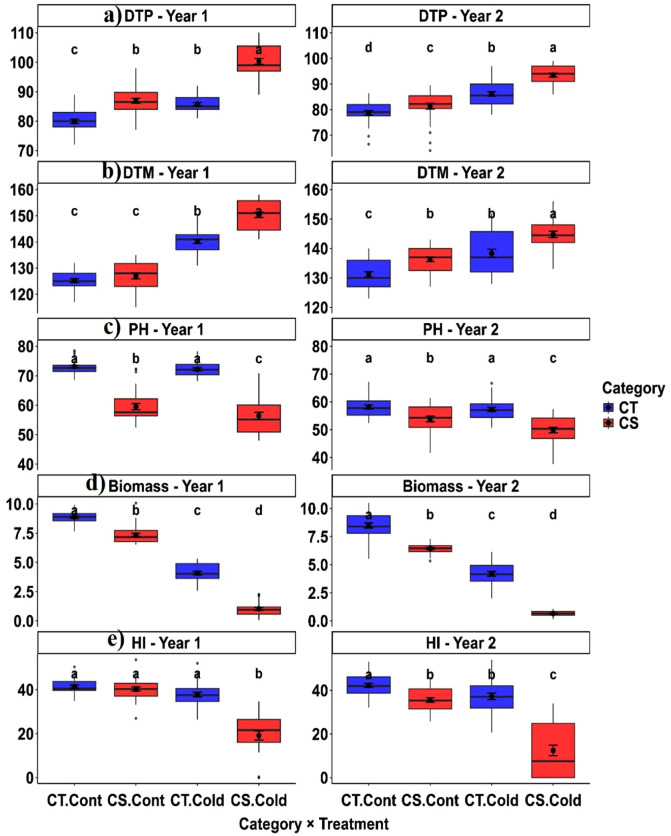
Comparison of phenological and growth traits between cold-tolerant (CT) and cold-sensitive (CS) chickpea genotypes under cold stress. Box plots represent (a) days to podding, (b) days to maturity, (c) plant height, (d) biomass, and (e) harvest index. Different letters indicate statistically significant differences between tolerance groups based on pairwise comparisons (P < 0.05). (Cont: Control).

The days to podding varied significantly across the genotype × treatment combinations (Fig 3a). Cold stress delayed podding by 6–8 days in CT genotypes and by 13–14 days in CS genotypes compared to their respective controls across both years, with a more pronounced delay in CS lines, suggesting greater sensitivity to low temperatures.

Days to maturity also showed considerable variation (Fig 3b). Cold stress delayed maturity by 12–15 days in CT genotypes and by 18–25 days in CS genotypes across both years. Among the phenological traits, days to maturity displayed pronounced genotypic variability and relatively higher heritability (Table 6), suggesting a greater potential for genetic improvement under cold stress.

**Table 6:** Summary statistics for key traits in selected chickpea genotypes under cold stress. (Trait units are as defined in Table 3).

Traits	Average		Range		Genotypic variance		Error		Heritability (%)	
	1^st^ year	2^nd^ year	1^st^ year	2^nd^ year	1^st^ year	2^nd^ year	1^st^ year	2^nd^ year	1^st^ year	2^nd^ year
*Phenological traits*										
DTF	74 ± 1.13	73 ± 0.89	70–78	67–78	0	0.14	16.1	18.3	0	0.7
DTP	93 ± 0.90	89 ± 0.25	84–107	80–98	5.13	10.6	13.7	10.0	27.1	51.3
DTM	145 ± 0.10	148 ± 0.58	136–155	129–154	14.4	23.2	12.0	24.2	54.4	48.9
*Growth traits*										
PH	64.2 ± 0.43	53.5 ± 0.2	48.6–76.1	38.3–65.4	19.5	21.5	6.7	3.00	74.2	87.7
Biomass	2.5 ± 0.07	2.42 ± 0.03	0.08–5.06	0.36–5.90	0.45	0.52	0.12	0.22	78.1	70.2
HI	28.4 ± 0.60	24.8 ± 1.54	0–41.4	0–44.1	39.0	83.7	43.8	36.4	0.47	69.7
*Physiological traits*										
EL	22.2 ± 0.87	21.6 ± 1.14	15.2–28.5	14.5–28.4	3.3	4.8	1.3	0.17	72.0	96.6
MDA	26.7 ± 1.11	26.6 ± 1.27	17.7–34.1	20.5–33.7	2.21	1.93	1.44	0.37	60.5	83.9
RLWC	79.6 ± 0.61	84.2 ± 0.95	70.6–87.7	80.1–87.8	17.1	3.66	2.65	2.04	86.8	64.3
SPAD	25.2 ± 0.56	17.4 ± 1.31	18.4–32.5	11.3–25.1	4.6	6.9	2.2	1.3	67.1	83.9
gS	63.2 ± 2.37	66.4 ± 1.28	33.6–98.4	36.5–89.2	144.5	35.2	30.7	8.50	82.4	80.5
ChlF	0.66 ± 0.02	0.6 ± 0.006	0.52–0.77	0.5–0.8	0.0004	0.002	0.0003	0.0003	79.4	85.1
NA	18.1 ± 0.92	19.0 ± 0.52	11.5–26.8	13.2–24.4	1.94	6.85	0.63	0.60	75.2	91.8
*Reproductive traits*										
PV	70.4 ± 2.16	69.4 ± 1.18	47.6–93	46.3–87.7	31.2	48.0	10.0	1.66	87.9	96.6
PG	61.5 ± 1.99	68.8 ± 1.11	35.3–88.6	50.7–84.7	25.5	36.9	13.1	3.5	81.0	91.1
SR	2.69 ± 0.22	2.8 ± 0.12	1–4.3	1–5	0.22	0.44	0.13	0.19	62.1	40.3
OV	3.01 ± 0.26	2.9 ± 0.14	1–4.3	1–5	0.20	0.15	0.16	0.18	56.6	44.9
*Antioxidants*										
SOD	21.6 ± 0.39	19.5 ± 0.91	11.5–27.1	15.5–24.4	3.58	1.85	1.74	0.16	67.2	91.8
CAT	4.69 ± 0.29	4.2 ± 0.29	3.21–6.52	2.2–6.3	0.5	0.13	0.019	0.01	96.5	92.4
APX	5.92 ± 0.22	6.03 ± 0.26	3.24–8.47	3.3–8.6	0.27	0.38	0.07	0.03	78.3	92.4
GR	5.60 ± 0.26	5.9 ± 0.23	3.16–8.24	3.6–9.3	0.36	1.34	0.015	0.05	96.0	95.9
AsA	139.4 ± 3.47	122.4 ± 2.36	106–178	102–154	111.8	58.0	3.54	11.7	96.9	83.1
GSH	28.1 ± 1.33	53.9 ± 1.78	12.6–42.6	40.5–73.8	17.9	36.0	0.45	0.86	97.5	97.6
Caro	3.04 ± 0.19	3.04 ± 0.16	2.14–4.27	2.2–4.3	0.075	0.26	0.06	0.10	53.3	72.6
*Osmolytes*										
Pro	22.6 ± 1.65	22.6 ± 1.56	15.4–29.8	14.6–32.5	1.02	9.29	2.03	0.23	33.3	97.5
Treh	3.61 ± 0.26	3.5 ± 0.18	2.22–5.32	1.4–5.5	0.12	0.57	0.03	0.05	80.2	90.7
TS	64.1 ± 1.50	74 ± 2.05	42.6–85.2	58.3–88.4	19.5	9.28	0.38	1.53	98.0	85.7
*Yield traits*										
FP	6.3 ± 0.22	6.4 ± 0.10	0–12	0–13	0.50	0.24	0.35	0.15	59.1	0.61
10sw	1.11 ± 0.01	1.09 ± 0.01	0–1.79	0–2.24	0.08	0.25	0.01	0.06	83.8	81.1
TSW	0.83 ± 0.05	0.8 ± 0.06	0–2.7	0–2.1	0.03	0.05	0.03	0.02	47.6	64.5
SN	6.61 ± 0.21	6.5 ± 0.14	0–13	0–14	0.80	0.33	0.38	0.29	67.8	53.6

**Abbreviations**: DTF: Days to flowering, DTP: Days to podding, DTM: days to maturity, PH: plant height, HI: harvest index, FP- filled pod; 10-SW: 10-seed weight; TSW-total seed weight; SN-seed number, EL, electrolyte leakage; MDA, malondialdehyde content; RLWC, relative leaf water content; SPAD, SPAD chlorophyll; gS, stomatal conductance; ChlF, chlorophyll fluorescence; NA, nodulation ability; SOD, superoxide dismutase; CAT, catalase; APX, ascorbate peroxidase; GR, glutathione reductase; AsA, Ascorbic acid; GSH, reduced glutathione; Caro, carotenoid; Pro, proline; TS, total sugars; Treh, trehalose; PV, pollen viability; PG, pollen germination; SR, stigma receptivity; OV, ovule viability.

#### 3.5.2. Growth traits.

Cold-tolerant genotypes consistently exhibited greater plant height than cold-sensitive genotypes under both control and cold stress conditions in both years (Fig 3c, Table 5). Growth traits exhibited high heritability and genotypic variance (Table 6).

Biomass accumulation showed clear advantages in the tolerant group (Fig 3d). CT genotypes maintained significantly greater aboveground biomass under stress across both years, reflecting enhanced vigour and biomass production capacity at low temperatures.

The harvest index was significantly higher in CT genotypes (Fig 3e). The CT-control and CT-cold consistently formed the highest statistical groups in both years, whereas CS-cold exhibited the lowest values. The marked HI reduction under cold stress in CS genotypes indicates inefficient assimilate allocation to reproductive structures.

#### 3.5.3. Physiological responses.

**Fig 4:**
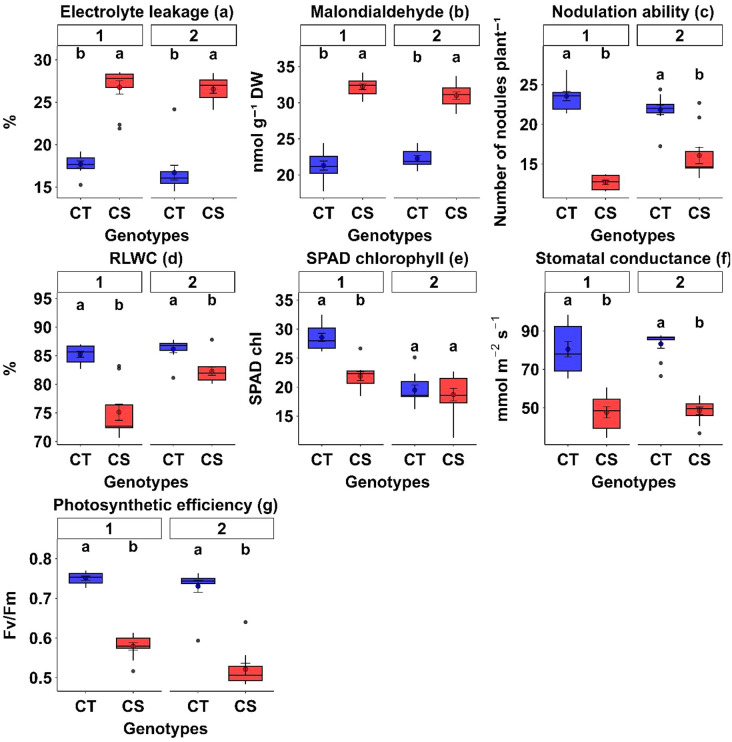
Comparison of physiological traits between cold-tolerant (CT) and cold-sensitive (CS) chickpea genotypes under cold stress. Box plots depict (a) electrolyte leakage, (b) malondialdehyde content, (c) nodulation ability, (d) relative leaf water content, **(e)** SPAD chlorophyll content, (f) stomatal conductance, and (g) photosynthetic efficiency for the first (left) and second (right) year. Distinct genotype sets were evaluated in each year; however, tolerant genotypes consistently exhibited reduced membrane damage (lower electrolyte leakage and MDA) and superior physiological performance. Different letters above the boxes indicate statistically significant differences between CT and CS groups within the same year, based on Tukey’s HSD test (P < 0.05).

Cold stress significantly increased electrolyte leakage (EL) and malondialdehyde (MDA) in both CT and CS genotypes, with CS genotypes showing greater increases (EL: 50–58.8% higher; MDA: 39–51.1% higher than CT across both years; Fig 4, Table 5, P < 0.01).

CT genotypes maintained healthier leaf physiology under cold stress, with higher relative leaf water content (4.5–10.9%), chlorophyll index (21.5–23.4%), stomatal conductance (40.1–42.6%), and photosynthetic efficiency (22.9–28.6%) than CS genotypes across both years (Fig 4, Table 5). Nodulation ability was also enhanced in CT genotypes (26.5–46.2% above CS genotypes).

Physiological traits exhibited high heritability across both years (Table 6), indicating strong genetic control and stability under cold stress.

#### 3.5.4. Reproductive traits.

**Fig 5:**
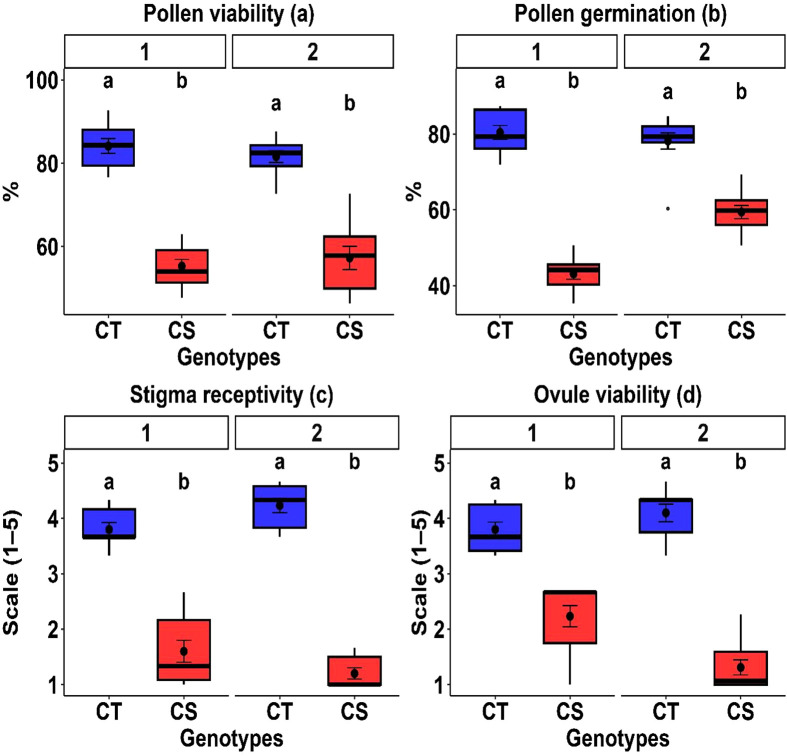
Comparison of floral traits between cold-tolerant (CT) and cold-sensitive (CS) chickpea genotypes under cold stress. Box plots represent (a) pollen viability, (b) pollen germination, (c) stigma receptivity, and (d) ovule viability for the first (left) and second (right) year. Distinct genotype sets were evaluated in each year, with tolerant genotypes consistently outperforming sensitive genotypes. Different letters above the boxes indicate statistically significant differences between CT and CS groups within the same year, based on Tukey’s HSD test (P < 0.05).

Cold stress significantly affected reproductive performance, with CT genotypes consistently outperforming CS genotypes (P < 0.01). Pollen viability and germination were 29.8–35.4% and 24–46% higher in CT genotypes, respectively, while stigma receptivity and ovule viability were 57.9–67.6% and 41.1–56.8% higher across both years, respectively (Table 5). Box plots (Fig 5) revealed that CT genotypes exhibited higher median values and narrower interquartile ranges, reflecting more stable reproductive function under cold stress.

Pollen viability and germination exhibited high genotypic variance and heritability, indicating strong genetic control, which is suitable for selection (Table 6). In contrast, stigma receptivity and ovule viability showed lower genotypic variance and moderate heritability, suggesting greater environmental influence.

#### 3.5.5. Antioxidant responses.

The CT genotypes exhibited significantly higher activities of catalase (38.2–55%), ascorbate peroxidase (48.4–49.7%), and glutathione reductase (45.3–45.8%) compared to the CS genotypes across both years (Table 5, P < 0.01). Interestingly, superoxide dismutase activity was consistently higher in the CS genotypes (27.0–29.8%), indicating distinct antioxidant strategies. Non-enzymatic antioxidants were also elevated in the CT genotypes; ascorbic acid increased by 24.6–30.4% and glutathione by 32–52.4%. Carotenoid content was significantly higher in CT genotypes (24.6–36.4%), reflecting enhanced photoprotective capacity.

**Fig 6:**
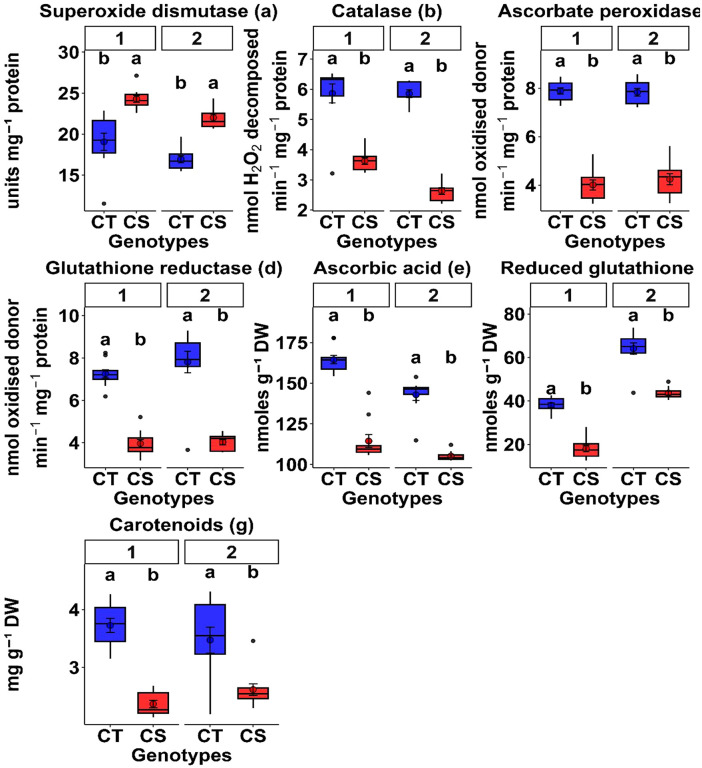
Comparison of antioxidant traits between cold-tolerant (CT) and cold-sensitive (CS) chickpea genotypes under cold stress. Box plots depict (a) superoxide dismutase (SOD), (b) catalase (CAT), (c) ascorbate peroxidase (APX), (d) glutathione reductase (GR), (e) ascorbic acid (AsA), (f) reduced glutathione (GSH), and (g) carotenoids (Caro) for the first (left) and second (right) year. Distinct genotype sets were evaluated in each year, with consistent response patterns across both years. Different letters above the boxes indicate statistically significant differences between CT and CS groups within the same year, based on Tukey’s HSD test (P < 0.05).

Box plots (Fig 6) demonstrate that CT genotypes consistently exhibited higher median values and lower variability for antioxidant traits. High heritability (Table 6) supports the stability of these traits across environments, making them valuable selection criteria for breeding programs.

#### 3.5.6. Osmolytes.

**Fig 7:**
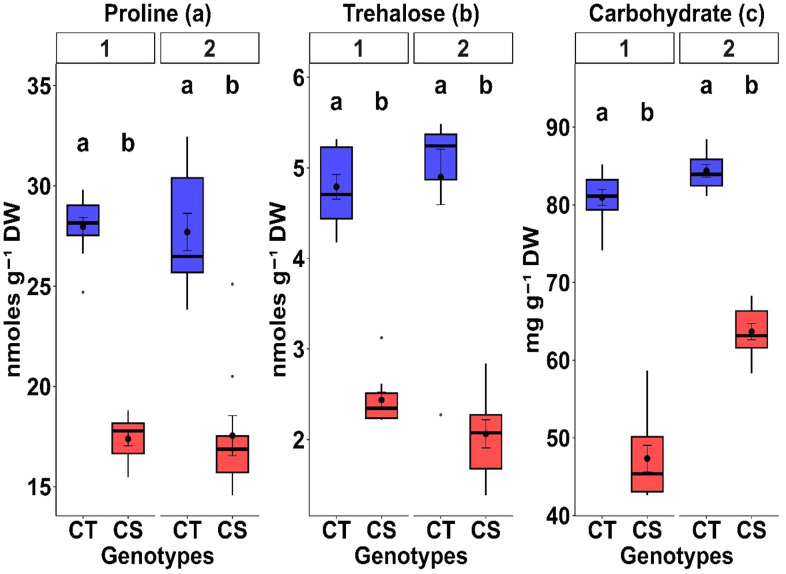
Comparison of cryoprotective traits between cold-tolerant (CT) and cold-sensitive (CS) chickpea genotypes under cold stress. Box plots show (a) proline, (b) trehalose, and (c) total sugars for the 1st (left) and 2nd (right) year. Distinct genotype sets were used, with tolerant genotypes consistently outperforming sensitive genotypes. Different letters above the boxes indicate statistically significant differences between CT and CS groups within the same year, based on Tukey’s HSD test (P < 0.05).

CT genotypes accumulated higher levels of proline (36.7–37.8%), trehalose (49.1–57.9%), and total sugars (24.5–41.5%) under cold stress than CS genotypes (Table 5, Fig 7, P < 0.01). These trends, coupled with their high heritability (Table 6), suggest that osmolytes and cryoprotectants are reliable physiological markers for selecting cold-tolerant chickpea genotypes.

#### 3.5.7. Yield traits.

**Fig 8:**
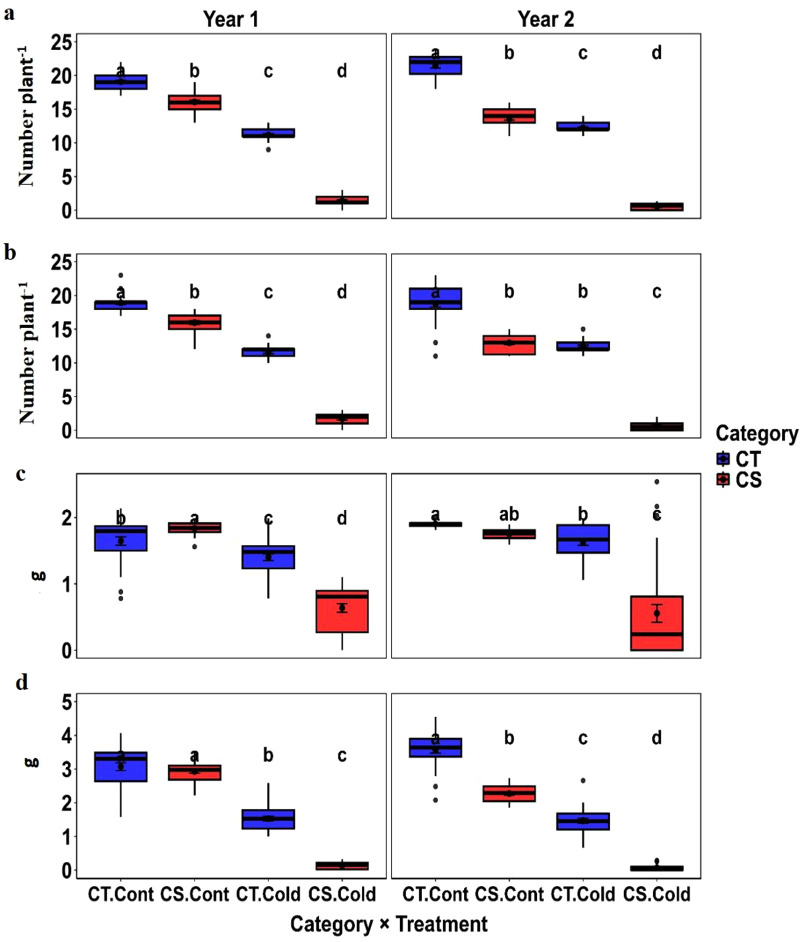
Comparison of the yield trait between cold-tolerant (CT) and cold-sensitive (CS) chickpea genotypes under cold stress. Box plots show (a) filled pods, (b) seed number, c) 10-seed weight, and **d)** Total seed weight. Distinct genotype sets were used each year, with tolerant genotypes consistently outperforming sensitive genotypes. Letters denote statistically significant differences between tolerance groups based on pairwise comparison (p < 0.05). (Cont: Control).

Compared to CS genotypes, CT genotypes exhibited substantially higher yield components under cold stress (Fig 8, Table 5). The CT genotypes showed 88.1–95.7% more filled pods, 54.5–65.9% higher 10-seed weight, 86.2–95.4% higher total seed weight, and 86.0–95.2% more seeds across both years.

Under control conditions, the differences were comparatively smaller; CT genotypes recorded 15–36.5% more filled pods, 7.8–11.4% higher 10-seed weight, 18–36% higher total seed weight, and 15–30% more seeds. High heritability and genotypic variance support the genetic stability of the yield traits (Table 6).

### 3.6. Principal component analysis and heat maps

PCA biplots revealed strong correlations among traits, with PC1 accounting for 81.3% (Year 1) and 72.6% (Year 2) of the cumulative variance, and PC2 contributing 4.2% and 5.6%, respectively, bringing the total explained variance to 85.5% and 78.2% (S8 Fig in S1 File). The arrow direction and length indicate the contribution magnitude of each variable, with the colour intensity representing the contribution strength (darker red indicates a higher contribution).

Across both years, PC1 consistently emerged as the major axis of variation, with most traits showing positive correlations. Exceptions included EL, MDA, SOD, DTP, DTM, and DTF, which had negative loadings, indicating inverse relationships with reproductive, yield-related, and other adaptive traits.

Hierarchical clustering using heat maps separated genotypes into two categories: CS genotypes, characterized by elevated EL, MDA, SOD, DTP, DTM, and DTF; and CT genotypes, distinguished by higher values in other physiological, biochemical, reproductive, and yield traits for both years (S9 Fig in S1 File).

### 3.7. Pearson correlation analysis

Pearson correlation analysis (S10 Fig in S1 File) was conducted separately for both years to complement the PCA and investigate trait relationships under cold stress. Key yield traits (FP, 10-SW, TSW, and SN) showed strong positive correlations with RLWC, ChlF, SPAD, Treh, Pro, TS, CAT, and APX (r up to 0.90, p < 0.001), while negatively correlating with MDA, EL, and SOD (r as low as −0.91). These patterns were consistent across both years, despite the different genotype sets, highlighting the physiological and biochemical traits most closely associated with yield performance under cold stress.

Biomass was positively correlated with phenology traits (DTF and DTM), indicating that late-flowering and late-maturing genotypes accumulate more vegetative mass owing to the extended growth duration. In contrast, the harvest index displayed weak or negative correlations with phenology, indicating that additional biomass did not translate into proportional seed yield increases. Thus, delayed phenology promotes vegetative growth but not reproductive efficiency, and early or optimal flowering is more favourable for achieving a higher harvest index under cold stress.

### 3.8. Radar plots

Radar plots (S11 Fig in S1 File) revealed consistent physiological and biochemical differences between the CT and CS genotypes in both years. CT genotypes maintained higher chlorophyll content, chlorophyll fluorescence, stomatal conductance, biomass, and reproductive traits (pod set, seed number, and total seed weight). Conversely, CS genotypes showed higher levels of stress injury markers (MDA and electrolyte leakage), together with reduced antioxidant activity and lower reproductive performance. The repeated patterns across years indicate that CT genotypes consistently sustain superior physiological functioning and lower oxidative damage under cold stress, whereas CS genotypes exhibit greater cellular injury and reduced yield-related traits under the same conditions.

## 4. Discussion

### 4.1. Phenology, biomass, and source-sink dynamics

Phenology showed weak associations with cold tolerance residuals (R² = 0.10–0.18), indicating that tolerance classification was independent of flowering time, which is an important finding given the potential phenology-linked confounding in stress studies. Cold stress delayed flowering and pod initiation, consistent with previous chickpea studies showing that chilling prolongs vegetative growth and disrupts pod set [48]. These delays are closely linked to biomass limitations, which reduce sink strength and yield potential [21]. Our findings indicate that maintaining developmental progression under stress, rather than phenological escape, is critical for cold tolerance in chickpea [19,49].

Cold stress caused extensive biomass reductions, but CT genotypes retained significantly higher biomass than CS lines. Reduced biomass reflects inhibited leaf expansion, impaired photosynthesis, and restricted assimilate transport [5,50], explaining why CT genotypes with superior photosynthetic functions retained more biomass. This biomass maintenance is critical because vegetative growth provides the source capacity necessary for the development of reproductive sinks. The strong positive associations between biomass ratio and cold tolerance residuals (R² = 0.51–0.66) demonstrate that genotypes capable of sustaining vegetative growth under stress exhibit superior tolerance, consistent with the source-sink framework described by Vadez et al. [21], in which limited early season growth restricts yield potential in chickpea.

The harvest index showed pronounced genotypic variation; CS lines exhibited severe reductions (often >50%, sometimes 100% due to complete seed abortion), whereas CT genotypes maintained smaller reductions (8–12%). This indicates superior assimilate partitioning toward reproductive structures in tolerant genotypes [50], demonstrating that tolerance reflects metabolic and reproductive resilience rather than simply plant size or phenological escape. The integration of biomass, harvest index, and phenology revealed that tolerance was not a function of any single growth parameter but reflected the coordinated physiological and reproductive processes. The biomass was positively correlated with phenological traits (days to flowering and maturity), indicating that late-flowering genotypes accumulated more vegetative mass owing to the extended growth duration. However, the harvest index displayed weak or negative correlations with phenology, demonstrating that additional biomass did not translate proportionally into seed yield. Thus, delayed phenology promotes vegetative growth but not reproductive efficiency, and optimum or slightly early flowering combined with high biomass accumulation capacity appears to be the most favourable for achieving a superior yield under cold stress.

### 4.2. Integrated physiological and biochemical defense mechanisms

Cold tolerance operates through the hierarchical integration of cellular protection mechanisms. Membrane stability (measured as electrolyte leakage) emerged as a foundational trait. CT genotypes maintained substantially lower electrolyte leakage (EL) and malondialdehyde (MDA; indicator of membrane damage)) levels, preserving the cellular compartmentation necessary for metabolic function. Membrane destabilization in CS genotypes, through cold-induced reductions in membrane fluidity [51] and accelerated ion efflux [52], compromises downstream physiological processes. This pattern aligns with observations in chickpea [6,53], rice [54], tomatoes [55], and lentils [56], establishing membrane protection as a universal requirement for chilling tolerance. The strong negative correlations between electrolyte leakage/MDA and all downstream traits (r = −0.89 to −0.91, p < 0.001) indicate that membrane integrity functions as the primary gatekeeper, enabling continued organellar function, metabolic homeostasis, and ultimately, reproductive success.

Building upon membrane stability, CT genotypes sustained higher chlorophyll content, PSII efficiency, stomatal conductance, and relative leaf water content, preserving photosynthetic function critical for continued carbon assimilation and energy supply [6]. This coordinated maintenance of water relations and photosynthetic capacity, observed across diverse species, including Stevia [57], sugarcane [58], and white clover [59], explains the superior biomass retention in tolerant genotypes. Maintaining chlorophyll content and efficient PSII activity is essential for sustaining photosynthesis under low temperatures, as cold stress typically impairs light reactions through its effects on thylakoid membrane fluidity, electron transport chain components, and carbon fixation enzymes. The higher stomatal conductance maintained in CT genotypes (40–43% above CS genotypes) facilitated continued CO₂ uptake for photosynthesis despite chilling conditions, directly supporting the observed maintenance of photosynthetic efficiency and carbohydrate production necessary for reproductive processes.

The enhanced nodulation ability in CT genotypes (26–47% above CS genotypes) suggests that membrane stability and metabolic homeostasis extend beyond shoot tissues to root-associated symbiotic processes. This finding is particularly significant because adequate nitrogen availability supports the synthesis of protective proteins (including antioxidant enzymes and stress-responsive proteins), amino acids (including proline), and reproductive structures during stress. The maintenance of nodule function under cold stress likely contributes to the superior nitrogen status of CT genotypes, supporting their enhanced metabolic capacity and reproductive development.

CT genotypes exhibited a coordinated cryoprotectant accumulation. Enhanced levels of proline, trehalose, and soluble sugars provide osmotic adjustment, stabilize membranes and proteins, and contribute to ROS scavenging [60–63]. This metabolic reprogramming, observed in chickpea [64], Brassica [65,66], sorghum [67] wheat [68], and other species, represents a conserved response to chilling stress that maintains cellular homeostasis necessary for continued metabolism and reproduction. The mechanistic rationale for coordinated cryoprotectant and antioxidant responses becomes clear when considering that cold stress simultaneously induces osmotic stress(through dehydration), membrane destabilization (through lipid phase transitions), and oxidative stress (through disrupted mitochondrial electron transport and impaired photosynthetic capacity). Therefore, a successful cold tolerance strategy must address all three challenges simultaneously rather than sequentially.

Cold stress induces oxidative stress through reactive oxygen species (ROS) accumulation and lipid peroxidation [69–71]. CT genotypes maintained lower MDA levels through enhanced enzymatic and non-enzymatic (ascorbate and glutathione) antioxidant activities. The coordinated upregulation of H₂O₂-scavenging enzymes (catalase and ascorbate peroxidase) and glutathione recycling (involving glutathione reductase) efficiently managed oxidative damage, consistent with patterns in chickpea [6,72], Brassica [73], rice [74,75], and wheat [76]. Interestingly, superoxide dismutase (SOD) activity was higher in CS genotypes (27–30%), suggesting that cold tolerance relies more on efficient downstream ROS scavenging than on preventing initial superoxide formation [74,77]. This elevated SOD level without corresponding increases in downstream H₂O₂-scavenging enzymes may exacerbate oxidative stress by converting superoxide to H₂O₂ faster than it can be removed, potentially leading to hydroxyl radical formation through Fenton reactions if H₂O₂ accumulates. This interpretation emphasizes that effective cold tolerance requires balanced and coordinated antioxidant responses rather than isolated enzyme induction.

The enhanced glutathione reductase activity in CT genotypes (45–46% above CS) maintains redox homeostasis by regenerating reduced glutathione from its oxidized form, thereby sustaining the cellular pool of this crucial antioxidant and supporting the ascorbate-glutathione cycle. This is particularly important for reproductive tissues, as a recent chickpea study reported marked GR increases in the anthers and ovules of tolerant genotypes under cold stress [72], highlighting its importance not only for redox balance but also in preserving reproductive function under low temperatures. Non-enzymatic antioxidants (ascorbate and glutathione) were significantly more abundant in the CT genotypes, providing additional buffering capacity and supporting the ascorbate-glutathione cycle, which is central to cellular ROS management.

Carotenoid content was also significantly higher in the CT genotypes (25–36% above CS levels), reflecting enhanced photoprotective capacity. Carotenoids function in light harvesting, dissipation of excess excitation energy through the xanthophyll cycle, quenching of singlet oxygen and triplet chlorophyll, and stabilization of photosynthetic membrane structures. The elevation in CT genotypes supports the observed maintenance of chlorophyll content and photosynthetic efficiency by protecting the photosynthetic apparatus from photo-oxidative damage.

Thus, the integrated responses in CT genotypes, membrane stability, water conservation, preserved photosynthesis, osmolyte accumulation, and higher antioxidant activity, provide a mechanistic explanation for improved cold resilience, consistent with observations in chickpea [8], *Brassica oleracea* [73], and several other species [14]. The high heritability of these physiological and biochemical traits (70–90% across most parameters) confirms their genetic stability and suitability for selection in breeding programs, indicating strong genetic control and a reliable response to selection pressure.

### 4.3. Reproductive resilience under cold stress

Reproductive resilience is the ultimate determinant of cold tolerance. Cold stress disrupts pollen formation, stigma function, and ovule viability, directly limiting the yield [19,49]. This reproductive vulnerability is consistent with that of legumes and cereals. In chickpea, CT genotypes sustain reproductive function through enhanced antioxidants and cryoprotectants [20]. Similar patterns have been observed in soybean (24% yield loss) [78] and sorghum (30% pollen viability reduction in sensitive lines) [79], confirming that reproductive-stage chilling represents a universal constraint on grain legumes and cereals. In rice, CT genotypes outperformed CS genotypes in reproductive success under cold stress, with tolerant lines showing maintained anther structure, higher pollen viability, and normal pollen tube growth [80]. In wheat, superior stigma receptivity under cold stress is correlated with improved fertilization rates and yield [50], demonstrating the importance of female reproductive function in addition to pollen viability.

CT genotypes maintained high pollen viability, germination, stigma receptivity, and ovule viability under chilling conditions, whereas CS genotypes exhibited severe reproductive failure, consistent with chickpea [6], rice [80], and sorghum [81] studies, demonstrating that reproductive organ protection is central to cold tolerance. These reproductive advantages were not independent traits but rather functional outcomes of the integrated physiological and biochemical protections operating at the whole-plant level. Our correlation analyses revealed that pollen viability was strongly positively associated with catalase activity (r = 0.88), proline content (r = 0.87), and chlorophyll fluorescence (r = 0.89), and negatively correlated with electrolyte leakage (r = −0.89) and MDA (r = −0.87). Similarly, ovule viability was positively correlated with trehalose (r = 0.86), total sugar (r = 0.84), and APX activity (r = 0.85). These relationships confirm that reproductive success under cold stress depends on integrated whole-plant stress defense mechanisms, including stable membranes providing proper cellular compartmentation, active photosynthesis supplying carbohydrates and energy, and effective antioxidant systems protecting reproductive tissues from oxidative damage, rather than reproductive-organ-specific adaptations operating in separation.

The strong positive correlations between reproductive traits and yield (r > 0.85, p < 0.001) confirm that cold tolerance is fundamentally determined by reproductive resilience. Genotypes that sustain pollen function, stigma-pollen interactions, and ovule viability through integrated physiological and biochemical protection demonstrate superior yield stability [19,49]. This explains why cold stress sharply separates CT from CS genotypes; once reproductive processes fail, yield potential collapses, regardless of vegetative health. Importantly, while CT and CS genotypes performed relatively similarly under control conditions (with CT genotypes showing only 8–37% advantages in yield traits), cold stress sharply separated the two groups; CT lines sustained 85–95% higher yields through maintained pod set, seed size, and seed number, whereas CS genotypes exhibited pronounced reproductive failure under identical stress conditions. These patterns demonstrate that cold stress greatly accentuates the inherent differences in tolerance mechanisms between the two groups and that the true value of cold tolerance traits becomes apparent only under stress conditions.

The heritability estimates for reproductive traits showed clear patterns, with pollen viability and germination exhibiting high heritability (81.0–96.6%) and substantial genotypic variance in both years, indicating strong genetic control suitable for selection. In contrast, stigma receptivity and ovule viability showed lower and more variable heritability (40.3–62.1% for stigma receptivity, 44.9–56.6% for ovule viability), with relatively low genotypic variance, suggesting greater environmental influence and lower reliability as direct selection traits. This pattern indicates that while all reproductive traits contribute to cold tolerance, pollen-related traits may serve as more reliable selection criteria in breeding programs because of their higher heritability and stability across environments.

### 4.4. Integrated tolerance networks in cold tolerance

Multivariate analysis revealed that cold tolerance operates as an integrated system. PCA showed that photosynthetic efficiency, water status, cryoprotectants, and antioxidants were positively correlated with yield, whereas EL and MDA were negatively correlated, confirming membrane stability as the foundational trait enabling downstream protection [10,50]. PC1, explaining 72.6–81.3% of the total variance, distinguished tolerant from sensitive genotypes, indicating that cold tolerance operates primarily as a systemic property rather than through multiple independent mechanisms. Most adaptive traits loaded positively on PC1, whereas stress damage indicators (EL, MDA, and SOD) loaded negatively, indicating inverse relationships with reproductive and yield-related traits.

Cluster and heat map analyses separated genotypes into distinct tolerance profiles. CT genotypes showed low oxidative injury, stable photosynthesis, and maintained reproduction, whereas CS genotypes exhibited system-wide disruption beginning with membrane damage cascading to metabolic and reproductive failure. These integrative patterns elucidate the sharp decline in yield observed in CS genotypes. Once membrane integrity is compromised, downstream physiological and metabolic processes fail to recover, leading to inevitable reproductive damage. Radar plots further illustrated that tolerant genotypes consistently maintained a balanced enhancement across physiological, antioxidant, and reproductive traits, whereas sensitive genotypes showed corresponding deficits across all functional categories.

These multivariate patterns demonstrate that cold tolerance is a multidimensional phenotype requiring coordinated membrane protection, metabolic adjustment, and reproductive resilience, highlighting the value of multi-trait selection over single-trait approaches for breeding applications. For practical breeding, electrolyte leakage measured at the reproductive stage (early flowering to pod set) provides a rapid and cost-effective selection criterion that requires only a conductivity meter and fresh leaf samples. As demonstrated by the clear separation of CT and CS genotypes in both heat map and PCA analyses across independent experiments, EL can effectively identify sensitive genotypes for elimination in early breeding generations. Combined with the assessment of photosynthetic efficiency (chlorophyll fluorescence or SPAD chlorophyll index) and subsequently pollen viability in advanced lines, this hierarchical screening approach maximizes efficiency by focusing labour-intensive reproductive assessments only on lines already shown to possess underlying physiological tolerance mechanisms.

To enhance breeding relevance, all 100 genotypes were classified into early-, medium-, and late-maturity groups. Within each maturity class, we identified cold-tolerant donor lines: ICC 516, ICC 43, ICC 216, ICC 621, ICC 2221, ICC 1437, ICC 1583, ICC 1881 from the early group; ICC 595, ICC 860, ICC 609, ICC 1925 from the medium group; and ICC 1881 from the late group. Identifying donors within specific maturity categories ensures that trait improvement can be aligned with target product profiles and duration requirements of different agroecological zones. This maturity-based donor identification enhances the applicability of our findings to breeding programs aimed at incorporating cold tolerance without altering crop duration, providing breeders with flexible genetic resources tailored to diverse production environments.

## 5. Conclusion and future perspectives

Cold stress during chickpea’s reproductive phase need not remain an intractable problem. By evaluating 200 genotypes across two independent experiments, we identified the lines that tolerate cold temperatures and the underlying mechanisms. Cold tolerance operates as an integrated hierarchical system, beginning with membrane stability, enabling metabolic reprogramming that protects photosynthesis, and sustains the biochemical machinery required for successful reproduction. Twenty consistently tolerant genotypes spanning different maturity classes provide breeders with flexible genetic resources for diverse production environments. The two-stage selection strategy we have outlined, rapid membrane and photosynthetic screening followed by targeted reproductive assessment, offers an efficient pathway for developing the cold-resilient cultivars needed across northern South Asia and beyond. With these tools and genetic resources, it may be feasible to improve chickpea productivity in cold-prone regions.

## Supporting information

S1 File Description of all supporting figures and tables included in the Supporting Information file. (DOCX)
