# Supplementary material for: Genotypic variability and trait associations for cold stress tolerance in cultivated chickpea (Cicer arietinum L.) during the reproductive stage
Source: PLoS One. 2026 Feb 26;21(2):e0343120. doi: 10.1371/journal.pone.0343120 (PMC12944791; doi:10.1371/journal.pone.0343120)
Supplement: S1 File — (DOCX) [file pone.0343120.s001.docx]

**Table S1. List of 100 chickpea genotypes (Set 1) evaluated for cold tolerance in the 1^st^ year, procured from the International Crops Research Institute for the Semi-Arid Tropics (ICRISAT), Hyderabad, India.**

| 1. ICC 12 | 16. ICC 434 | 31. ICC 595 | 46. ICC 780 | 61. ICC 922 | 76. ICC1039 | 91. ICC 1270 |
| --- | --- | --- | --- | --- | --- | --- |
| 2. ICC 43 | 17. ICC 438 | 32. ICC 599 | 47. ICC 781 | 62. ICC 927 | 77. ICC 1101 | 92. ICC 1271 |
| 3. ICC 121 | 18. ICC 444 | 33. ICC 607 | 48. ICC 801 | 63. ICC 933 | 78. ICC 1129 | 93. ICC 1279 |
| 4. ICC 154 | 19. ICC 449 | 34. ICC 609 | 49. ICC 805 | 64. ICC 958 | 79. ICC 1132 | 94. ICC 1283 |
| 5. 1CC 181 | 20. ICC 460 | 35. ICC 610 | 50. ICC 816 | 65. ICC 959 | 80. ICC 1149 | 95. ICC 1292 |
| 6. ICC 182 | 21. ICC 461 | 36. ICC 613 | 51. ICC 843 | 66. ICC 967 | 81. ICC 1155 | 96. ICC 1294 |
| 7. ICC 202 | 22. ICC 478 | 37. ICC 621 | 52. ICC 857 | 67. ICC 999 | 82. ICC 1159 | 97. ICC 1298 |
| 8. ICC 209 | 23. ICC 516 | 38. ICC 645 | 53. ICC 858 | 68. ICC 1003 | 83. ICC 1170 | 98. ICC 1314 |
| 9. ICC 216 | 24. ICC 519 | 39. ICC 647 | 54. ICC 859 | 69. ICC 1005 | 84. ICC 1179 | 99. ICC 1316 |
| 10. ICC 240 | 25. ICC 535 | 40. ICC 657 | 55. ICC 860 | 70. ICC 1012 | 85. ICC 1202 | 100. ICC 1317 |
| 11. ICC 250 | 26. ICC 537 | 41. ICC 658 | 56. ICC 880 | 71. ICC 1016 | 86. ICC 1209 |  |
| 12. ICC 267 | 27. ICC 554 | 42. ICC 665 | 57. ICC 884 | 72. ICC 1017 | 87. ICC 1212 |  |
| 13. ICC 268 | 28. ICC 573 | 43. ICC 739 | 58. ICC 902 | 73. ICC 1019 | 88. ICC 1214 |  |
| 14. ICC 301 | 29. ICC 585 | 44. ICC 758 | 59. ICC 903 | 74. ICC 1023 | 89. ICC 1234 |  |
| 15. ICC 391 | 30. ICC 594 | 45. ICC 773 | 60. ICC 904 | 75. ICC 1038 | 90. ICC 1250 |  |

**Table S2. List of 100 chickpea genotypes (Set 2) evaluated for cold tolerance in the 2^nd^ year, procured from the International Crops Research Institute for the Semi-Arid Tropics (ICRISAT), Hyderabad, India.**

| 1. ICC 1319 | 16. ICC 1451 | 31. ICC 1624 | 46. ICC 1731 | 61. ICC 1899 | 76. ICC 2039 | 91. ICC 2246 |
| --- | --- | --- | --- | --- | --- | --- |
| 2. ICC 1330 | 17. ICC 1467 | 32. ICC 1649 | 47. ICC 1749 | 62. ICC 1901 | 77. ICC 2041 | 92. ICC 2253 |
| 3. ICC 1352 | 18. ICC 1468 | 33. ICC 1663 | 48. ICC 1753 | 63. ICC 1902 | 78. ICC 2061 | 93. ICC 2264 |
| 4. ICC 1361 | 19. ICC 1477 | 34. ICC 1664 | 49. ICC 1755 | 64. ICC 1910 | 79. ICC 2089 | 94. ICC 2266 |
| 5. ICC 1376 | 20. ICC 1491 | 35. ICC 1669 | 50. ICC 1756 | 65. ICC 1918 | 80. ICC 2117 | 95. ICC 2267 |
| 6. ICC 1393 | 21. ICC 1504 | 36. ICC 1671 | 51. ICC 1758 | 66. ICC 1925 | 81. ICC 2156 | 96. ICC 2278 |
| 7. ICC 1395 | 22. ICC 1525 | 37. ICC 1686 | 52. ICC 1795 | 67. ICC 1939 | 82. ICC 2160 | 97. ICC 2286 |
| 8. ICC 1403 | 23. ICC 1532 | 38. ICC 1688 | 53. ICC 1809 | 68. ICC 1984 | 83. ICC 2165 | 98. ICC 2294 |
| 9. ICC 1434 | 24. ICC 1567 | 39. ICC 1694 | 54. ICC 1820 | 69. ICC 1987 | 84. ICC 2172 | 99. ICC 2295 |
| 10. ICC 1435 | 25. ICC 1583 | 40. ICC 1711 | 55. ICC 1826 | 70. ICC 2019 | 85. ICC 2199 | 100. ICC 2304 |
| 11. ICC 1437 | 26. ICC 1587 | 41. ICC 1712 | 56. ICC 1827 | 71. ICC 2031 | 86. ICC 2217 |  |
| 12. ICC 1441 | 27. ICC 1597 | 42. ICC 1716 | 57. ICC 1828 | 72. ICC 2032 | 87. ICC 2221 |  |
| 13. ICC 1448 | 28. ICC 1599 | 43. ICC 1718 | 58. ICC 1870 | 73. ICC 2034 | 88. ICC 2237 |  |
| 14. ICC 1449 | 29. ICC 1606 | 44. ICC 1719 | 59. ICC 1871 | 74. ICC 2036 | 99. ICC 2239 |  |
| 15. ICC 1450 | 30. ICC 1607 | 45. ICC 1723 | 60. ICC 1881 | 75. ICC 2037 | 90. ICC 2243 |  |

**Table S3: Summary of environmental conditions during different growth stages.**

| Stage | Location / Setup | Temperature (Day/Night, °C) | Light Conditions | Relative Humidity (%) | Notes |
| --- | --- | --- | --- | --- | --- |
| Sowing → Floral bud stage | Outdoor (natural conditions) | Max: **30–32 °C→15–18 °C**;  Min: **14–16 °C→ 5–7** **°C** (gradual seasonal cooling) | 1300–1500 μmol m⁻² s⁻¹ | 60–70 | Gradual cooling without abrupt cold exposure |
| 78 DAS after sowing | Plants were transferred to growth chambers | **25/15 °C** (12 h/12 h) → gradual reduction | 600 μmol m⁻² s⁻¹ | 65–70 | All genotypes had initiated flowering |
| Acclimation phase | Growth chamber | **25/15 °C** (12 h/12 h) → gradual reduction | 600 μmol m⁻² s⁻¹ | 65–70 | Gradual pre-cold acclimation |
| Cold stress phase | Growth chamber | **15/7 °C** (12 h/12 h) | 600 μmol m⁻² s⁻¹ | 65–70 | Controlled reproductive-stage cold stress was imposed for 15 days |
| Recovery phase | Growth chamber | **30/25 °C** | 600 μmol m⁻² s⁻¹ | 65–70 | Gradual recovery by 2 °C until physiological maturity |
| Maturity phase | Growth chamber | **30/25 °C** | 600 μmol m⁻² s⁻¹ | 65–70 | Warm conditions for pod-filling and maturity |
| Control (flowering → maturity) | Growth chamber | Constant **25/15 °C for 15 days**, then **30/25 °C** for maturity | 600 μmol m⁻² s⁻¹ | 65–70 | No cold stress applied; maintained throughout |

**Table S4: List of genotypes categorised on the basis of maturity under cold stress (Year 1)**

| Genotypes | Days to flowering | Days to maturity (cold) | Maturity group | Group |
| --- | --- | --- | --- | --- |
| ICC 516 | 73 | 136 | Early | Cold-tolerant |
| ICC 121 | 75 | 147 | Early | Moderately Cold-tolerant |
| ICC 621 | 73 | 137 | Early | Cold-tolerant |
| ICC 959 | 72 | 144 | Early | Moderately Cold-tolerant |
| ICC 250 | 70 | 147 | Early | Moderately Cold-tolerant |
| ICC 12 | 75 | 147 | Early | Moderately Cold-tolerant |
| ICC 1017 | 78 | 143 | Early | Cold-sensitive |
| ICC 216 | 74 | 140 | Early | Cold-tolerant |
| ICC 43 | 70 | 136 | Early | Cold-tolerant |
| ICC 780 | 76 | 146 | Early | Moderately Cold-tolerant |
| ICC 657 | 76 | 146 | Early | Moderately Cold-tolerant |
| ICC 958 | 72 | 145 | Early | Cold-sensitive |
| ICC 610 | 72 | 144 | Early | Cold-tolerant |
| ICC 858 | 70 | 143 | Early | Cold-tolerant |
| ICC 478 | 72 | 145 | Early | Cold-tolerant |
| ICC 519 | 75 | 147 | Early | Moderately Cold-tolerant |
| ICC 554 | 73 | 147 | Early | Moderately Cold-tolerant |
| ICC 460 | 74 | 147 | Early | Cold-sensitive |
| ICC 1279 | 77 | 142 | Early | Cold-sensitive |
| ICC 1005 | 74 | 145 | Early | Cold-sensitive |
| ICC 1214 | 77 | 144 | Early | Cold-sensitive |
| ICC 1003 | 75 | 143 | Early | Cold-sensitive |
| ICC 1016 | 77 | 142 | Early | Cold-sensitive |
| ICC 1101 | 76 | 144 | Early | Cold-sensitive |
| ICC 1212 | 77 | 145 | Early | Cold-sensitive |
| ICC 1023 | 74 | 145 | Early | Cold-sensitive |
| ICC 1271 | 76 | 141 | Early | Cold-sensitive |
| ICC 1019 | 78 | 140 | Early | Cold-sensitive |
| ICC 1149 | 78 | 145 | Early | Cold-sensitive |
| ICC 1129 | 76 | 143 | Early | Cold-sensitive |
| ICC 1132 | 73 | 145 | Early | Cold-sensitive |
| ICC 1298 | 73 | 147 | Early | Cold-sensitive |
| ICC 1012 | 76 | 142 | Early | Cold-sensitive |
| ICC 202 | 73 | 148 | Medium | Moderately Cold-tolerant |
| ICC 267 | 72 | 152 | Medium | Moderately Cold-tolerant |
| ICC 860 | 72 | 150 | Medium | Cold-tolerant |
| ICC 595 | 72 | 149 | Medium | Cold-tolerant |
| ICC 209 | 75 | 148 | Medium | Moderately Cold-tolerant |
| ICC 391 | 75 | 152 | Medium | Moderately Cold-tolerant |
| ICC 857 | 75 | 148 | Medium | Cold-sensitive |
| ICC 301 | 72 | 152 | Medium | Cold-sensitive |
| ICC 801 | 73 | 152 | Medium | Moderately Cold-tolerant |
| ICC 240 | 73 | 149 | Medium | Moderately Cold-tolerant |
| ICC 1292 | 73 | 151 | Medium | Cold-sensitive |
| ICC 665 | 73 | 152 | Medium | Moderately Cold-tolerant |
| ICC 573 | 74 | 149 | Medium | Moderately Cold-tolerant |
| ICC 607 | 78 | 150 | Medium | Moderately Cold-tolerant |
| ICC 1159 | 74 | 151 | Medium | Cold-sensitive |
| ICC 537 | 75 | 149 | Medium | Moderately Cold-tolerant |
| ICC 609 | 73 | 148 | Medium | Cold-tolerant |
| ICC 438 | 73 | 152 | Medium | Cold-sensitive |
| ICC 599 | 74 | 151 | Medium | Cold-sensitive |
| ICC 1294 | 74 | 149 | Medium | Cold-sensitive |
| ICC 1250 | 76 | 149 | Medium | Cold-sensitive |
| ICC 1283 | 77 | 149 | Medium | Cold-sensitive |
| ICC 1179 | 76 | 151 | Medium | Cold-sensitive |
| ICC 444 | 75 | 152 | Medium | Cold-sensitive |
| ICC 461 | 77 | 151 | Medium | Cold-sensitive |
| ICC 268 | 73 | 150 | Medium | Cold-sensitive |
| ICC 1209 | 76 | 149 | Medium | Cold-sensitive |
| ICC 1270 | 77 | 150 | Medium | Cold-sensitive |
| ICC 585 | 73 | 149 | Medium | Cold-sensitive |
| ICC 805 | 72 | 152 | Medium | Cold-sensitive |
| ICC 1316 | 74 | 152 | Medium | Cold-sensitive |
| ICC 1170 | 77 | 151 | Medium | Cold-sensitive |
| ICC 1155 | 76 | 150 | Medium | Cold-sensitive |
| ICC 182 | 75 | 153 | Late | Moderately Cold-tolerant |
| ICC 154 | 72 | 155 | Late | Moderately Cold-tolerant |
| ICC 773 | 73 | 155 | Late | Moderately Cold-tolerant |
| ICC 884 | 74 | 160 | Late | Moderately Cold-tolerant |
| ICC 880 | 70 | 160 | Late | Moderately Cold-tolerant |
| ICC 1038 | 76 | 156 | Late | Moderately Cold-tolerant |
| ICC 449 | 71 | 153 | Late | Moderately Cold-tolerant |
| ICC 613 | 72 | 153 | Late | Moderately Cold-tolerant |
| ICC 645 | 78 | 155 | Late | Moderately Cold-tolerant |
| ICC 816 | 73 | 153 | Late | Moderately Cold-tolerant |
| ICC 658 | 72 | 153 | Late | Moderately Cold-tolerant |
| ICC 933 | 74 | 155 | Late | Cold-sensitive |
| ICC 843 | 78 | 153 | Late | Moderately Cold-tolerant |
| ICC 647 | 73 | 154 | Late | Moderately Cold-tolerant |
| ICC 781 | 73 | 154 | Late | Cold-sensitive |
| ICC 999 | 74 | 155 | Late | Moderately Cold-tolerant |
| ICC 758 | 73 | 155 | Late | Moderately Cold-tolerant |
| 1CC 181 | 73 | 154 | Late | Cold-sensitive |
| ICC 594 | 77 | 159 | Late | Moderately Cold-tolerant |
| ICC 434 | 75 | 153 | Late | Cold-sensitive |
| ICC 903 | 73 | 157 | Late | Cold-sensitive |
| ICC 1039 | 78 | 153 | Late | Cold-sensitive |
| ICC 1314 | 76 | 155 | Late | Cold-sensitive |
| ICC 535 | 73 | 153 | Late | Cold-sensitive |
| ICC 922 | 75 | 155 | Late | Cold-sensitive |
| ICC 1202 | 77 | 154 | Late | Cold-sensitive |
| ICC 859 | 78 | 155 | Late | Cold-sensitive |
| ICC 1234 | 77 | 153 | Late | Cold-sensitive |
| ICC 967 | 74 | 153 | Late | Cold-sensitive |
| ICC 739 | 72 | 153 | Late | Cold-sensitive |
| ICC 904 | 75 | 157 | Late | Cold-sensitive |
| ICC 927 | 74 | 154 | Late | Cold-sensitive |
| ICC 902 | 70 | 158 | Late | Cold-sensitive |
| ICC 1317 | 77 | 154 | Late | Cold-sensitive |

**Table S5: List of genotypes categorized on the basis of maturity under cold stress (Year 2)**

| **Genotypes** | **Days to flowering** | **Days to maturity (cold)** | **Maturity group** | **Group** |
| --- | --- | --- | --- | --- |
| ICC 2221 | 66 | 129 | Early | Cold-tolerant |
| ICC 1437 | 71 | 134 | Early | Cold-tolerant |
| ICC 1583 | 65 | 134 | Early | Cold-tolerant |
| ICC 1403 | 68 | 130 | Early | Cold-tolerant |
| ICC 2036 | 72 | 135 | Early | Cold-sensitive |
| ICC 1435 | 67 | 137 | Early | Cold-tolerant |
| ICC 1567 | 74 | 140 | Early | Moderately Cold-tolerant |
| ICC 1532 | 76 | 140 | Early | Moderately Cold-tolerant |
| ICC 2034 | 67 | 140 | Early | Moderately Cold-tolerant |
| ICC 1984 | 67 | 133 | Early | Moderately Cold-tolerant |
| ICC 1624 | 73 | 138 | Early | Moderately Cold-tolerant |
| ICC 2286 | 76 | 139 | Early | Cold-sensitive |
| ICC 1731 | 77 | 138 | Early | Cold-sensitive |
| ICC 1319 | 73 | 138 | Early | Moderately Cold-tolerant |
| ICC 1663 | 72 | 138 | Early | Cold-sensitive |
| ICC 1525 | 73 | 136 | Early | Moderately Cold-tolerant |
| ICC 2264 | 76 | 134 | Early | Cold-sensitive |
| ICC 1352 | 71 | 132 | Early | Moderately Cold-tolerant |
| ICC 1330 | 75 | 135 | Early | Moderately Cold-tolerant |
| ICC 1491 | 78 | 136 | Early | Cold-sensitive |
| ICC 2041 | 67 | 134 | Early | Cold-sensitive |
| ICC 2089 | 67 | 135 | Early | Cold-sensitive |
| ICC 2165 | 67 | 140 | Early | Cold-sensitive |
| ICC 1664 | 71 | 139 | Early | Cold-sensitive |
| ICC 2117 | 67 | 137 | Early | Cold-sensitive |
| ICC 1910 | 74 | 135 | Early | Cold-sensitive |
| ICC 1987 | 67 | 134 | Early | Cold-sensitive |
| ICC 2253 | 71 | 133 | Early | Cold-sensitive |
| ICC 1649 | 75 | 140 | Early | Cold-sensitive |
| ICC 1686 | 77 | 139 | Early | Cold-sensitive |
| ICC 1711 | 77 | 135 | Early | Cold-sensitive |
| ICC 2237 | 76 | 134 | Early | Cold-sensitive |
| ICC 2061 | 67 | 133 | Early | Cold-sensitive |
| ICC 1395 | 70 | 138 | Early | Cold-sensitive |
| ICC 1749 | 74 | 143 | Medium | Cold-tolerant |
| ICC 1925 | 67 | 143 | Medium | Cold-tolerant |
| ICC 1449 | 76 | 144 | Medium | Moderately Cold-tolerant |
| ICC 1441 | 74 | 144 | Medium | Moderately Cold-tolerant |
| ICC 1712 | 74 | 145 | Medium | Moderately Cold-tolerant |
| ICC 1504 | 73 | 144 | Medium | Moderately Cold-tolerant |
| ICC 1587 | 74 | 143 | Medium | Moderately Cold-tolerant |
| ICC 1758 | 78 | 145 | Medium | Cold-sensitive |
| ICC 1716 | 77 | 144 | Medium | Moderately Cold-tolerant |
| ICC 1597 | 73 | 141 | Medium | Moderately Cold-tolerant |
| ICC 1448 | 78 | 143 | Medium | Cold-tolerant |
| ICC 1918 | 67 | 145 | Medium | Moderately Cold-tolerant |
| ICC 1755 | 77 | 145 | Medium | Cold-sensitive |
| ICC 2243 | 74 | 141 | Medium | Cold-sensitive |
| ICC 1434 | 76 | 142 | Medium | Moderately Cold-tolerant |
| ICC 2267 | 74 | 141 | Medium | Cold-sensitive |
| ICC 2019 | 74 | 142 | Medium | Cold-sensitive |
| ICC 1826 | 77 | 145 | Medium | Moderately Cold-tolerant |
| ICC 1467 | 69 | 142 | Medium | Cold-sensitive |
| ICC 2039 | 77 | 143 | Medium | Cold-sensitive |
| ICC 2278 | 77 | 141 | Medium | Cold-sensitive |
| ICC 2246 | 76 | 142 | Medium | Cold-sensitive |
| ICC 1450 | 73 | 144 | Medium | Cold-sensitive |
| ICC 2172 | 67 | 143 | Medium | Cold-sensitive |
| ICC 1393 | 75 | 143 | Medium | Cold-sensitive |
| ICC 1756 | 73 | 144 | Medium | Cold-sensitive |
| ICC 2032 | 74 | 144 | Medium | Cold-sensitive |
| ICC 1599 | 73 | 142 | Medium | Cold-sensitive |
| ICC 1753 | 77 | 141 | Medium | Cold-tolerant |
| ICC 1477 | 77 | 142 | Medium | Cold-sensitive |
| ICC 1451 | 70 | 145 | Medium | Cold-sensitive |
| ICC 1606 | 71 | 141 | Medium | Cold-sensitive |
| ICC 2156 | 65 | 141 | Medium | Cold-sensitive |
| ICC 1881 | 70 | 149 | Late | Cold-tolerant |
| ICC 1809 | 76 | 147 | Late | Moderately Cold-tolerant |
| ICC 1795 | 73 | 148 | Late | Moderately Cold-tolerant |
| ICC 1723 | 71 | 151 | Late | Cold-sensitive |
| ICC 1719 | 72 | 147 | Late | Cold-sensitive |
| ICC 1361 | 74 | 146 | Late | Cold-sensitive |
| ICC 1671 | 77 | 148 | Late | Cold-sensitive |
| ICC 1871 | 67 | 148 | Late | Cold-sensitive |
| ICC 2239 | 70 | 150 | Late | Cold-sensitive |
| ICC 1688 | 74 | 147 | Late | Moderately Cold-tolerant |
| ICC 1827 | 72 | 150 | Late | Cold-sensitive |
| ICC 2304 | 75 | 146 | Late | Cold-sensitive |
| ICC 1669 | 73 | 149 | Late | Cold-sensitive |
| ICC 2294 | 76 | 148 | Late | Cold-sensitive |
| ICC 1870 | 77 | 148 | Late | Moderately Cold-tolerant |
| ICC 1468 | 71 | 147 | Late | Cold-sensitive |
| ICC 1820 | 74 | 148 | Late | Cold-sensitive |
| ICC 2217 | 67 | 150 | Late | Cold-sensitive |
| ICC 1939 | 76 | 148 | Late | Cold-sensitive |
| ICC 2037 | 77 | 146 | Late | Cold-sensitive |
| ICC 1902 | 70 | 149 | Late | Cold-sensitive |
| ICC 1828 | 76 | 150 | Late | Cold-sensitive |
| ICC 1607 | 73 | 152 | Late | Cold-sensitive |
| ICC 1901 | 76 | 150 | Late | Cold-sensitive |
| ICC 1694 | 77 | 147 | Late | Moderately Cold-tolerant |
| ICC 1718 | 74 | 149 | Late | Cold-sensitive |
| ICC 2266 | 77 | 148 | Late | Cold-sensitive |
| ICC 2199 | 76 | 146 | Late | Cold-sensitive |
| ICC 1899 | 73 | 150 | Late | Cold-sensitive |
| ICC 2031 | 76 | 147 | Late | Cold-sensitive |
| ICC 1376 | 77 | 154 | Late | Cold-sensitive |
| ICC 2295 | 65 | 151 | Late | Cold-sensitive |
| ICC 2160 | 77 | 146 | Late | Cold-sensitive |

**Table S6:** **Regression values (R²) and significance of associations (P) between residual seed yield and physiological, reproductive, antioxidant, and osmolytes ratios under cold stress and control conditions for both years.**

| **Traits** | **1^st^ year** | | **2^nd^ year** | |
| --- | --- | --- | --- | --- |
|  | **R^2^** | **P value** | **R^2^** | **P value** |
| ***Physiological traits*** | | | | |
| **EL** | 0.19 | *** | 0.23 | *** |
| **MDA** | 0.21 | *** | 0.26 | *** |
| **RLWC** | 0.33 | *** | 0.25 | *** |
| **SPAD** | 0.21 | *** | 0.17 | *** |
| **gS** | 0.25 | *** | 0.24 | *** |
| **Chl. F** | 0.2 | *** | 0.26 | *** |
| **NA** | 0.15 | *** | 0.26 | *** |
| ***Reproductive traits*** | | | | |
| **PV** | 0.21 | *** | 0.28 | *** |
| **PG** | 0.35 | *** | 0.3 | *** |
| **SR** | 0.38 | *** | 0.35 | *** |
| **OV** | 0.33 | *** | 0.36 | *** |
| ***Antioxidants*** | | | | |
| **SOD** | 0.19 | *** | 0.2 | *** |
| **CAT** | 0.23 | *** | 0.18 | *** |
| **APX** | 0.21 | *** | 0.27 | *** |
| **GR** | 0.2 | *** | 0.33 | *** |
| **AsA** | 0.25 | *** | 0.24 | *** |
| **GSH** | 0.18 | *** | 0.25 | *** |
| **Caro** | 0.17 | *** | 0.22 | *** |
| ***Osmolytes*** | | | | |
| **PRO** | 0.21 | *** | 0.27 | *** |
| **TREH** | 0.14 | *** | 0.26 | *** |
| **TS** | 0.2 | *** | 0.25 | *** |

**Abbreviations**: EL, electrolyte leakage; MDA, malondialdehyde content; RLWC, relative leaf water content; SPAD, SPAD chlorophyll; gS, stomatal conductance; ChlF, chlorophyll fluorescence; NA, nodulation ability; SOD, superoxide dismutase; CAT, catalase; APX, ascorbate peroxidase; GR, glutathione reductase; AsA, Ascorbic acid; GSH, reduced glutathione; Caro, carotenoid; Pro, proline; TS, total sugars; Treh, trehalose; PV, pollen viability; PG, pollen germination; SR, stigma receptivity; OV, ovule viability. Asterisks indicate statistical significance: *** p < 0.001.


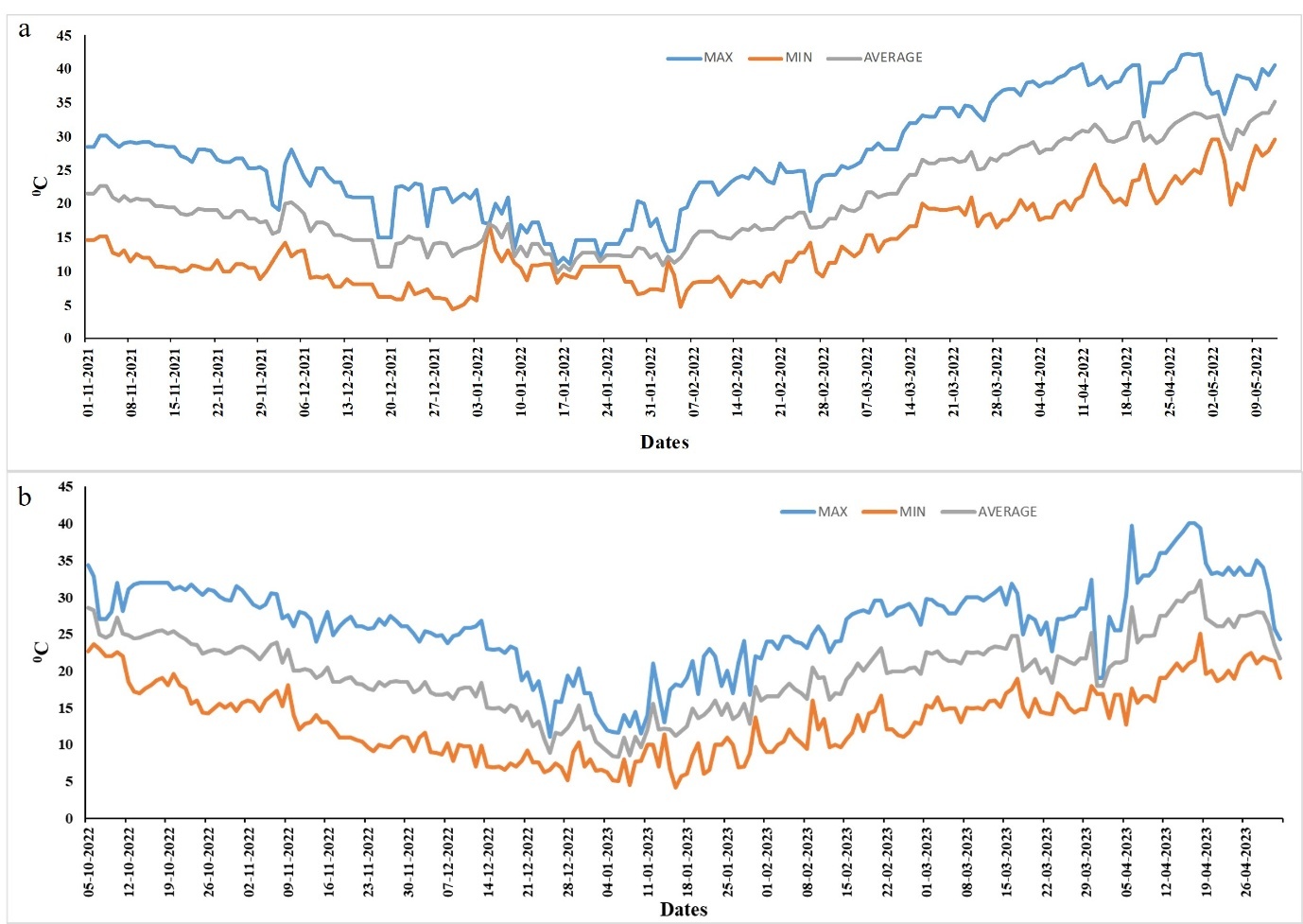


**Figure S1a.** Weather data showing maximum, minimum, and average temperatures from sowing to maturity for both years.


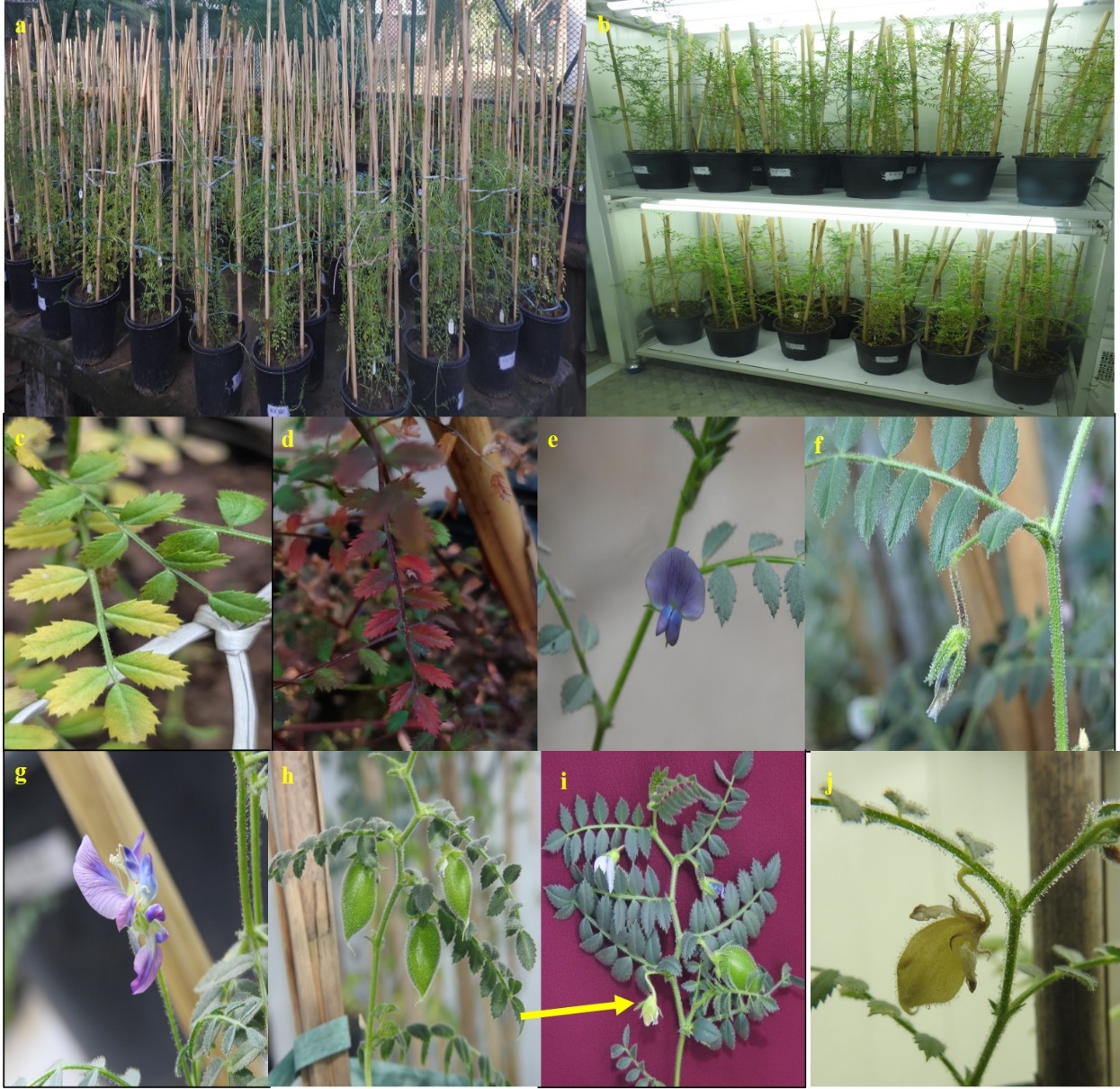


**Figure S1b.** Experimental area and phenotypic observations: (a) outdoor conditions, (b) growth chamber, (c) chlorosis, (d) anthocyanin accumulation, (e) healthy flower, (f) aborted flower, (g) exposed anthers, (h) healthy pods, (i) aborted pod, (j) unfilled pod.


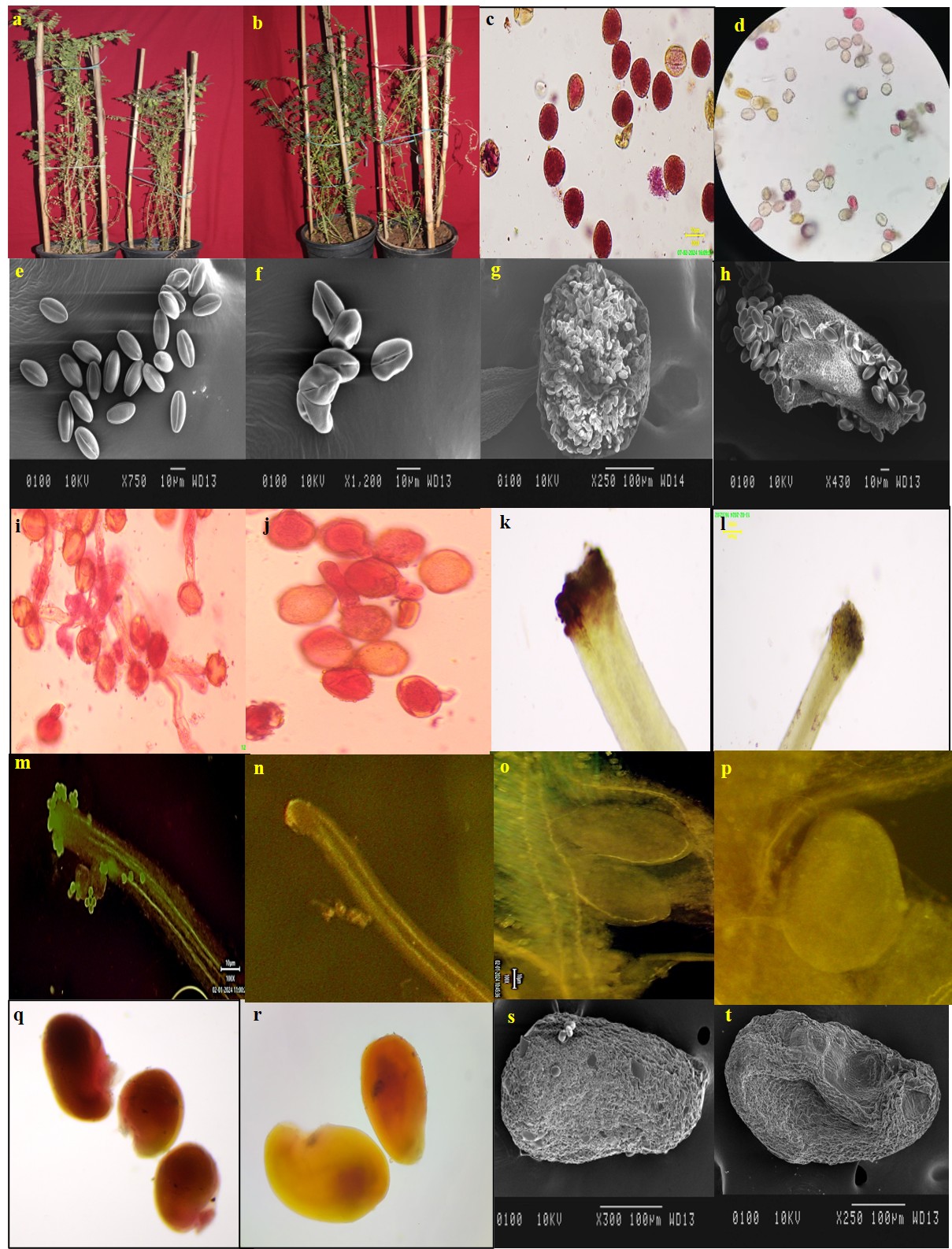


**Figure S1c.** Effects of cold stress on floral biology in contrasting chickpea genotypes (left: cold-tolerant (CT), right: cold-sensitive (CS)): (a, b) plant growth, (c, d) pollen viability (viable in CT, non-viable in CS), (e, f) SEM analysis of healthy (CT) and unhealthy (CS) pollen, (i, j) pollen gemination (higher in CT, lower in CS), (k, l) stigma receptivity (higher in CT, lower in CS), fluorescence microscopy showing (m, n) pollen load on stigma (higher in CT, lower or none in CS) and (o, p) in-vivo pollen tube growth in ovules (none in CS), (q, r) ovule viability (higher in CT, lower in CS, (s, t) ovules (healthy in CT, distorted in CS).

**
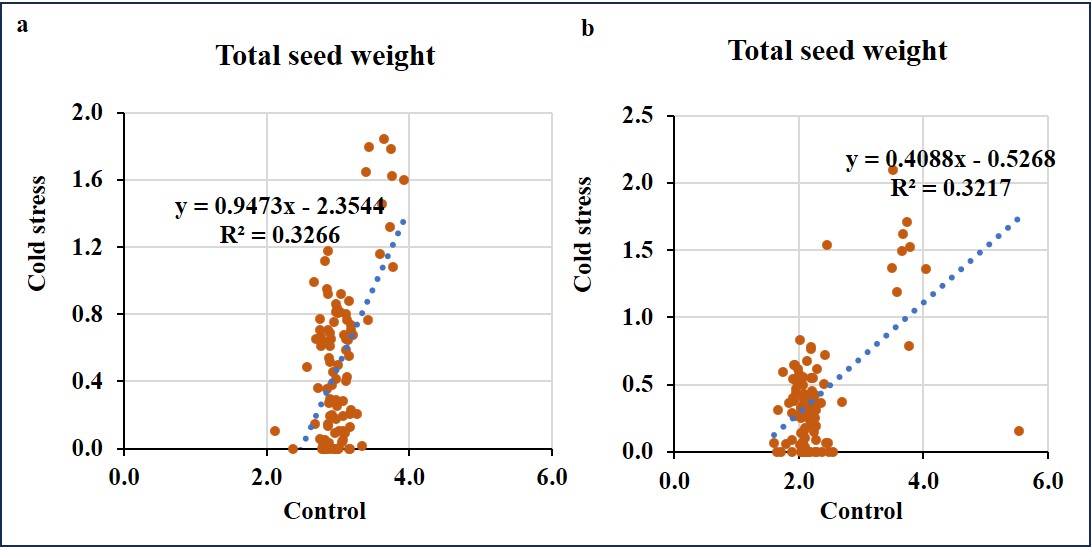
**

**Figure S2:** Relationship between seed yield in the control and seed yield under cold conditions for the a) 1^st^ year and b) 2^nd^ year.


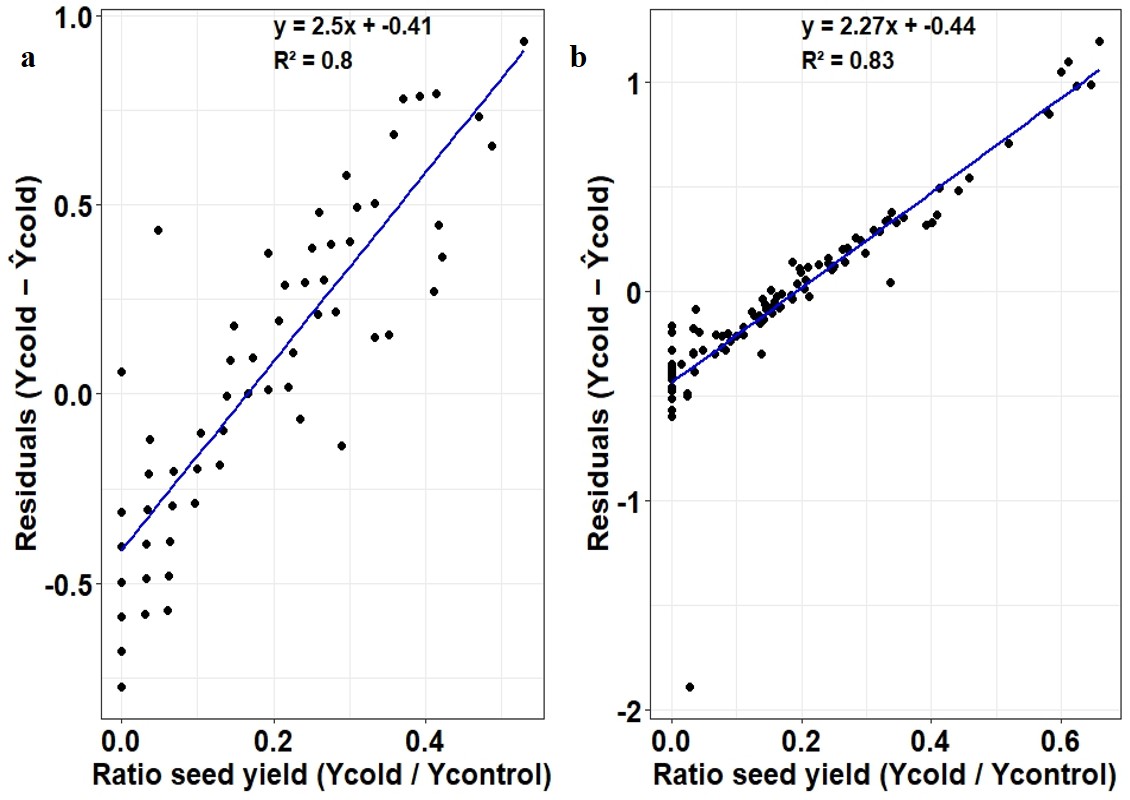


**Figure S3.** Regression between the ratio of seed yield under cold stress relative to control (Ycold/Ycontrol) and the residuals of seed yield (Ycold − Ŷcold), where Ŷcold represents the predicted cold yield based on yield potential for a) 1^st^ year and b) 2^nd^ year.


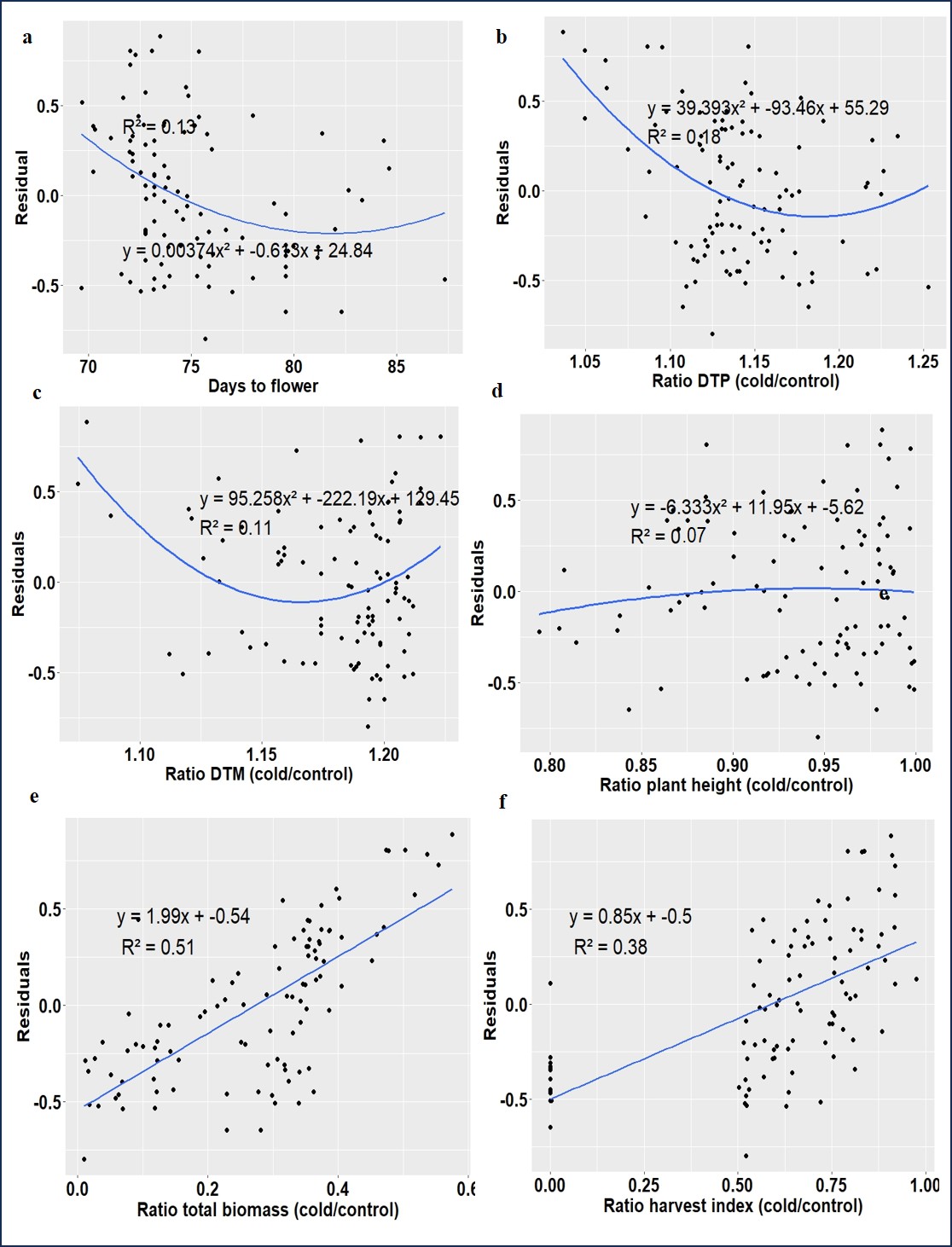


**Figure S4.** Regression analyses showing the association between cold-tolerance residuals (Ys – Ŷs) and phenology and growth traits under cold stress: (a) days to flowering, (b) days to podding (DTP), (c) days to maturity (DTM), d) plant height, (e) total biomass, and f) harvest index, for 1^st^ year.


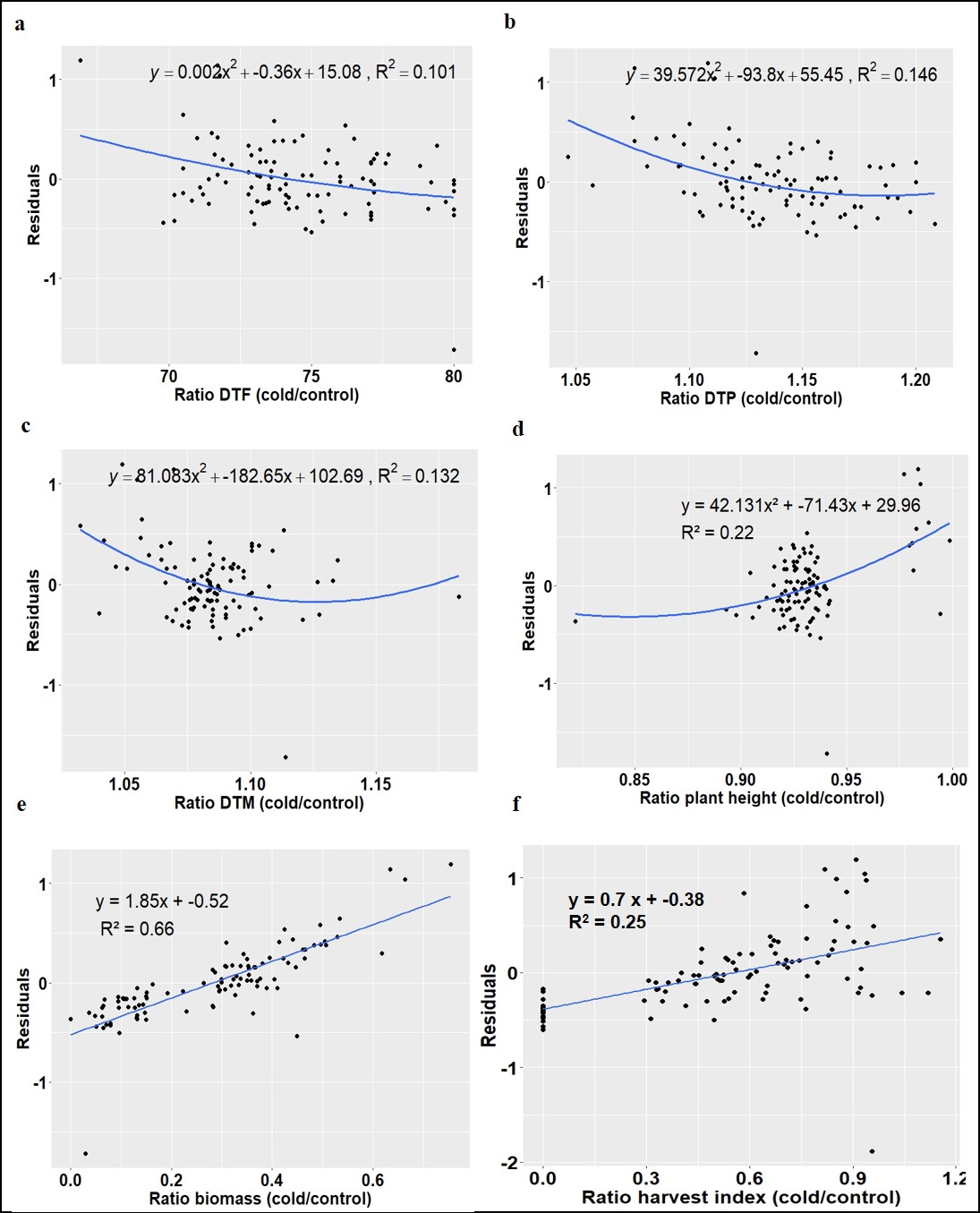


**Figure S5.** Regression analyses showing the association between cold-tolerance residuals (Ys – Ŷs) and phenology and growth traits under cold stress: (a) days to flowering, (b) days to podding (DTP), (c) days to maturity (DTM), d) plant height, (e) total biomass, and f) harvest index, for 2^nd^ year.

**
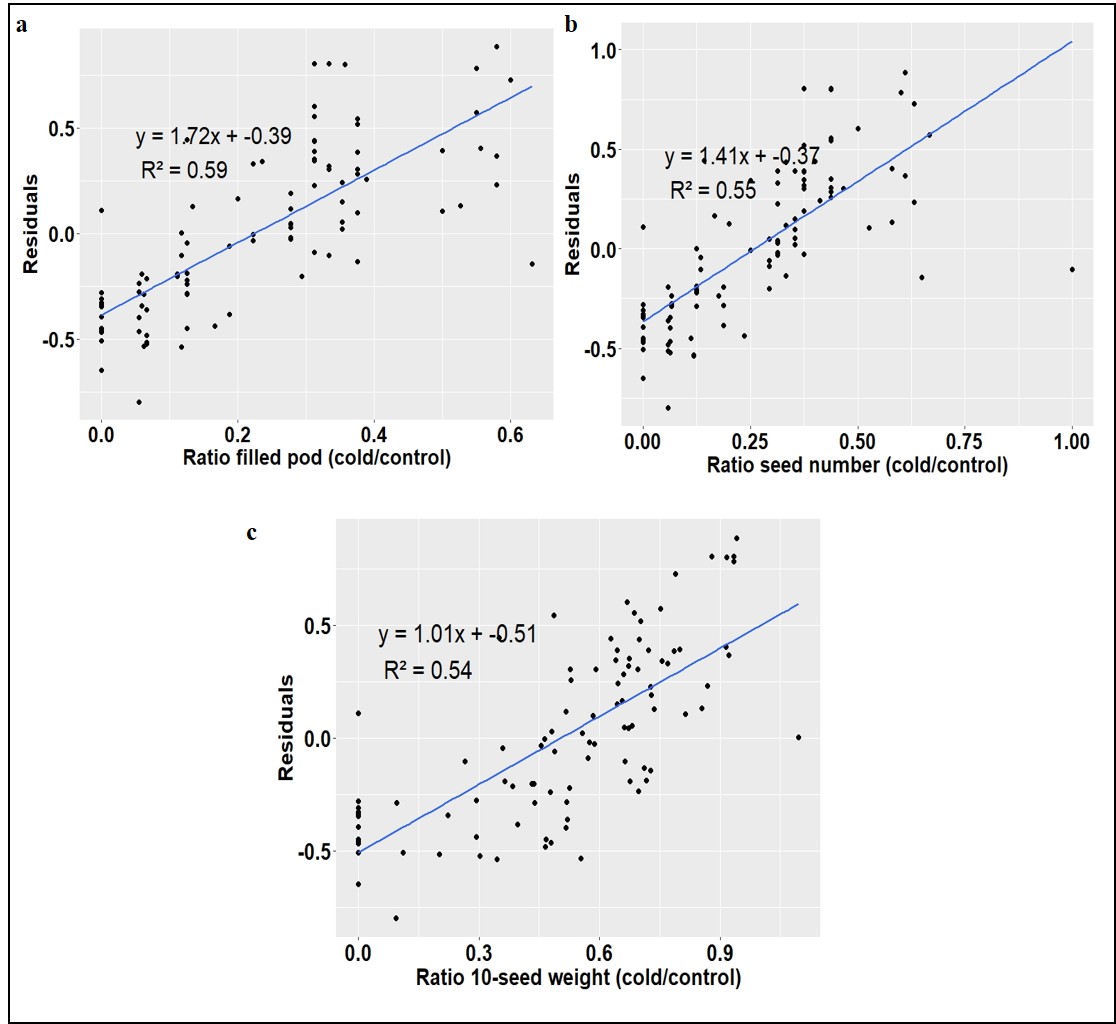
**

**Figure S6:** Regression analyses showing the association between cold-tolerance residuals (Ys – Ŷs) and major yield-related traits under cold stress: (a) filled pod ratio, (b) seed number ratio, (c) 10-seed weight ratio for 1^st^ year.


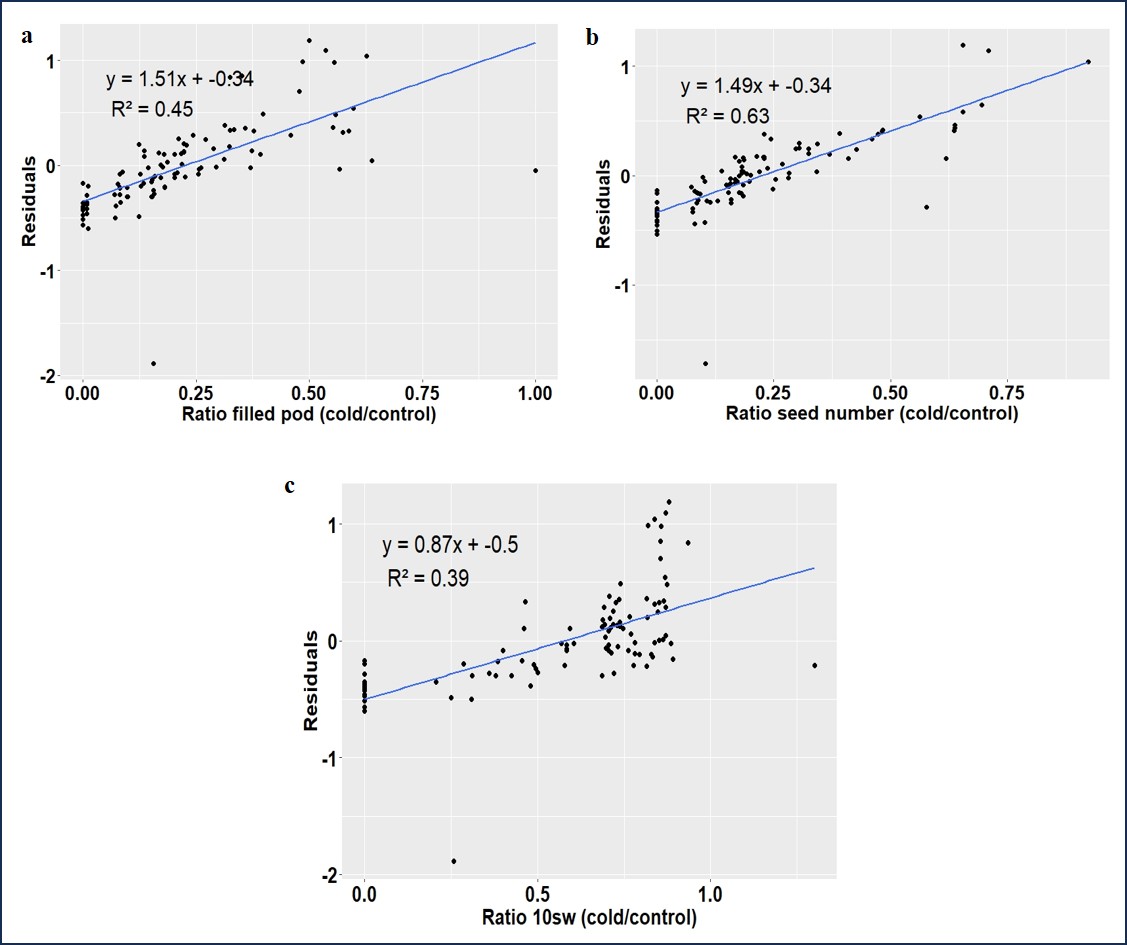


**Figure S7:** Regression analyses showing the association between cold-tolerance residuals (Ys – Ŷs) and major yield-related traits under cold stress: (a) filled pod ratio, (b) seed number ratio, (c) 10-seed weight ratio for the 2^nd^ year.


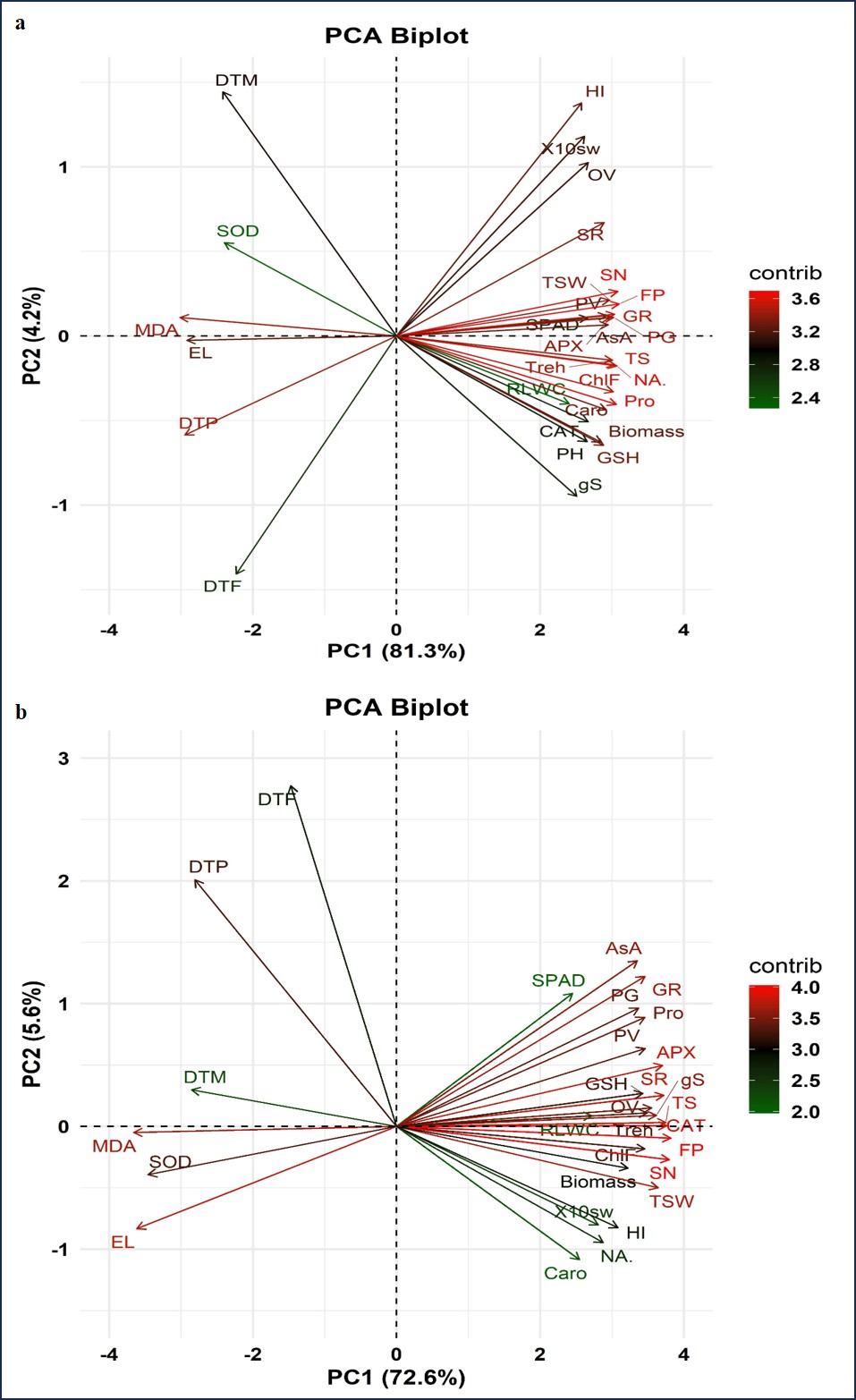


**Figure S8. (a)** PCA plot (Year 1) and **(b)** PCA (Year 2) showing growth traits, phenological traits, physiological traits, biochemical traits, reproductive and yield-related variables in cold-stressed chickpea genotypes. **Abbreviation**: PH: plant height; DTF: days to flowering, DTP: days to podding, DTM: days to maturity; HI: harvest index; RLWC: relative leaf water content, gS: stomatal conductance; SPAD: SPAD chlorophyll; Caro: carotenoid; Chl. F: chlorophyll fluorescence; NA: nodulation ability; EL: electrolyte leakage; MDA: malondialdehyde content; SOD: superoxide dismutase; CAT: catalase; APX: ascorbate peroxidase; GR: glutathione reductase; AsA: ascorbic acid; GSH: reduced glutathione; PV: pollen viability; PG: pollen germination, SR: stigma receptivity; OV: ovule viability; Pro: proline; TS; total sugars; Treh: trehalose, FP: filled pods; 10-sw: 10-seed weight; TSW: total seed weight; SN: seed number.


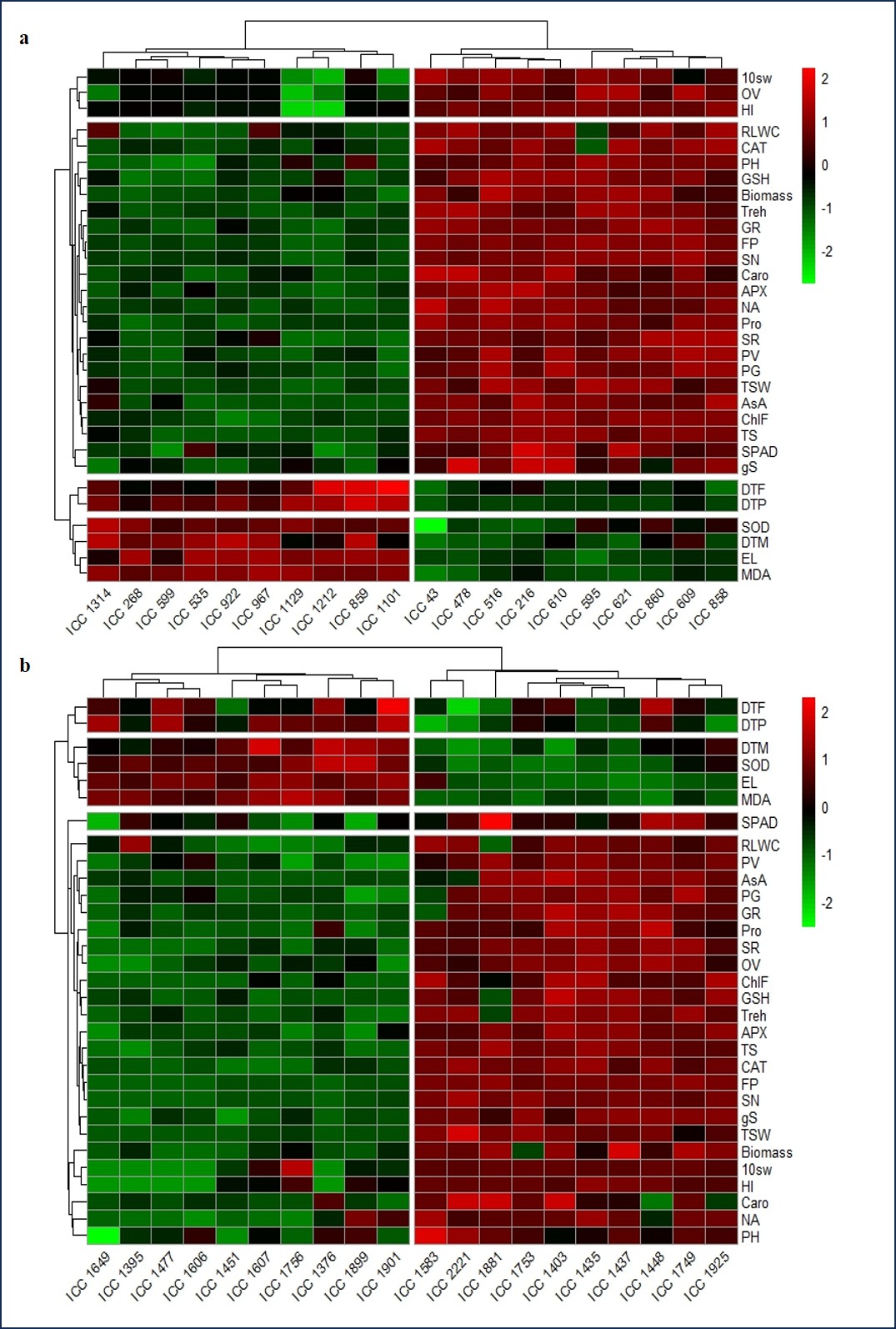


**Figure S9: (a) Heat map (Year 1)** and **(b)** heatmap (Year 2) showing growth traits, phenological traits, physiological traits, biochemical traits, reproductive and yield-related variables in cold-stressed chickpea genotypes. PH: plant height; DTF: days to flowering, DTP: days to podding, DTM: days to maturity; HI: harvest index; RLWC: relative leaf water content, gS: stomatal conductance; SPAD: SPAD chlorophyll; Caro: carotenoid; Chl. F: chlorophyll fluorescence; NA: nodulation ability; EL: electrolyte leakage; MDA: malondialdehyde content; SOD: superoxide dismutase; CAT: catalase; APX: ascorbate peroxidase; GR: glutathione reductase; AsA: ascorbic acid; GSH: reduced glutathione; PV: pollen viability; PG: pollen germination, SR: stigma receptivity; OV: ovule viability; Pro: proline; TS; total sugars; Treh: trehalose, FP: filled pods; 10-sw: 10-seed weight; TSW: total seed weight; SN: seed number. Colour: Red = higher standardised values (positive z-score), Green = lower standardized values (negative z-score), Black = mean/zero value. X-axis = genotypes (names must be readable), Y-axis = traits/parameters


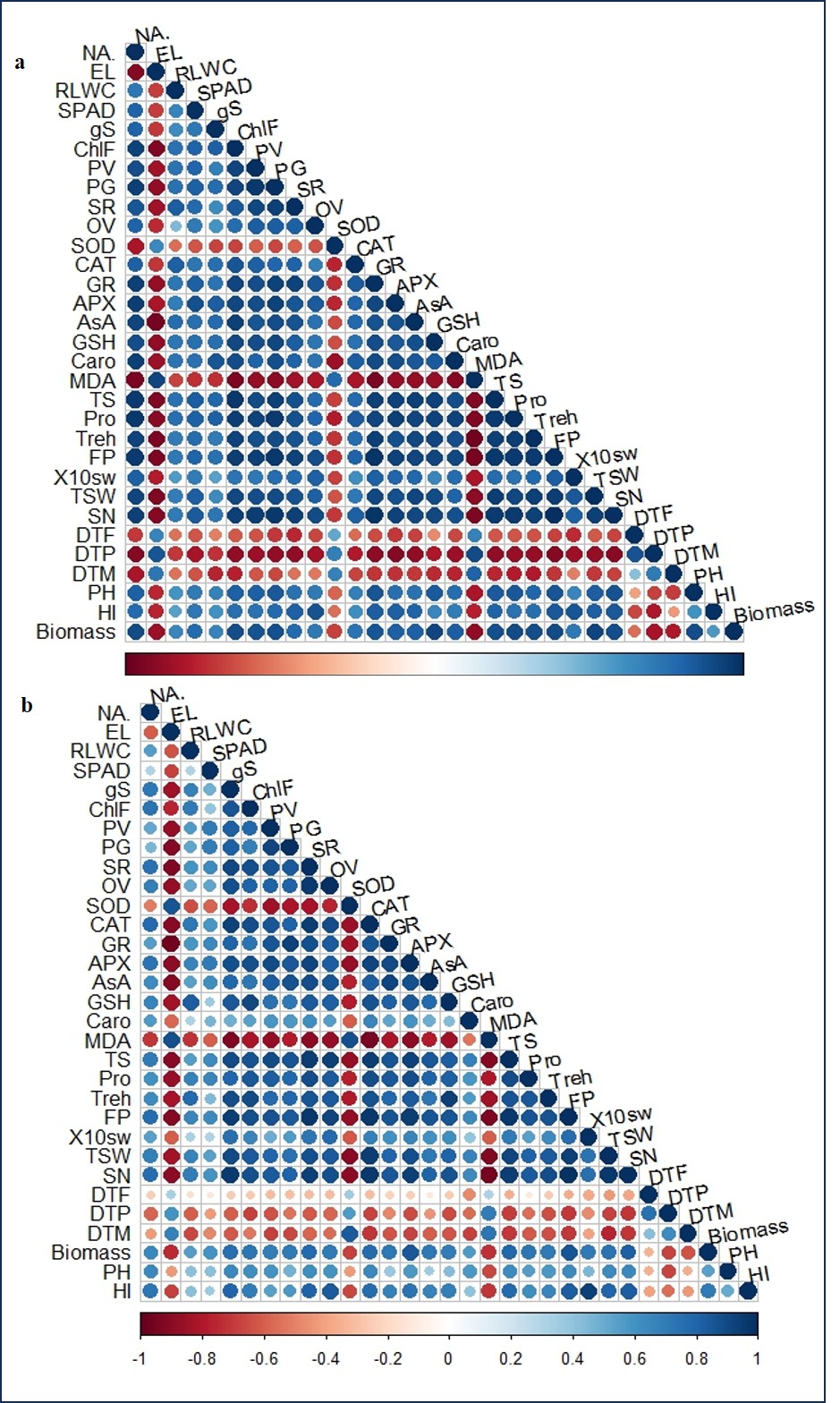


**Figure S10**: Pearson correlation plots (a) Year 1 and (b) Year 2, showing growth traits, phenological traits, physiological traits, biochemical traits, reproductive and yield-related variables in cold-stressed chickpea genotypes. The colour scale represents Pearson correlation coefficients, where blue indicates positive correlations, red indicates negative correlations, and colour intensity corresponds to the strength of the correlation (-1 to +1). Abbreviations: PH: plant height; DTF: days to flowering, DTP: days to podding, DTM: days to maturity; HI: harvest index; RLWC: relative leaf water content, gS: stomatal conductance; SPAD: SPAD chlorophyll; Caro: carotenoid; Chl. F: chlorophyll fluorescence; NA: nodulation ability; EL: electrolyte leakage; MDA: malondialdehyde content; SOD: superoxide dismutase; CAT: catalase; APX: ascorbate peroxidase; GR: glutathione reductase; AsA: ascorbic acid; GSH: reduced glutathione; PV: pollen viability; PG: pollen germination, SR: stigma receptivity; OV: ovule viability; Pro: proline; TS; total sugars; Treh: trehalose, FP: filled pods; 10-sw: 10-seed weight; TSW: total seed weight; SN: seed number.


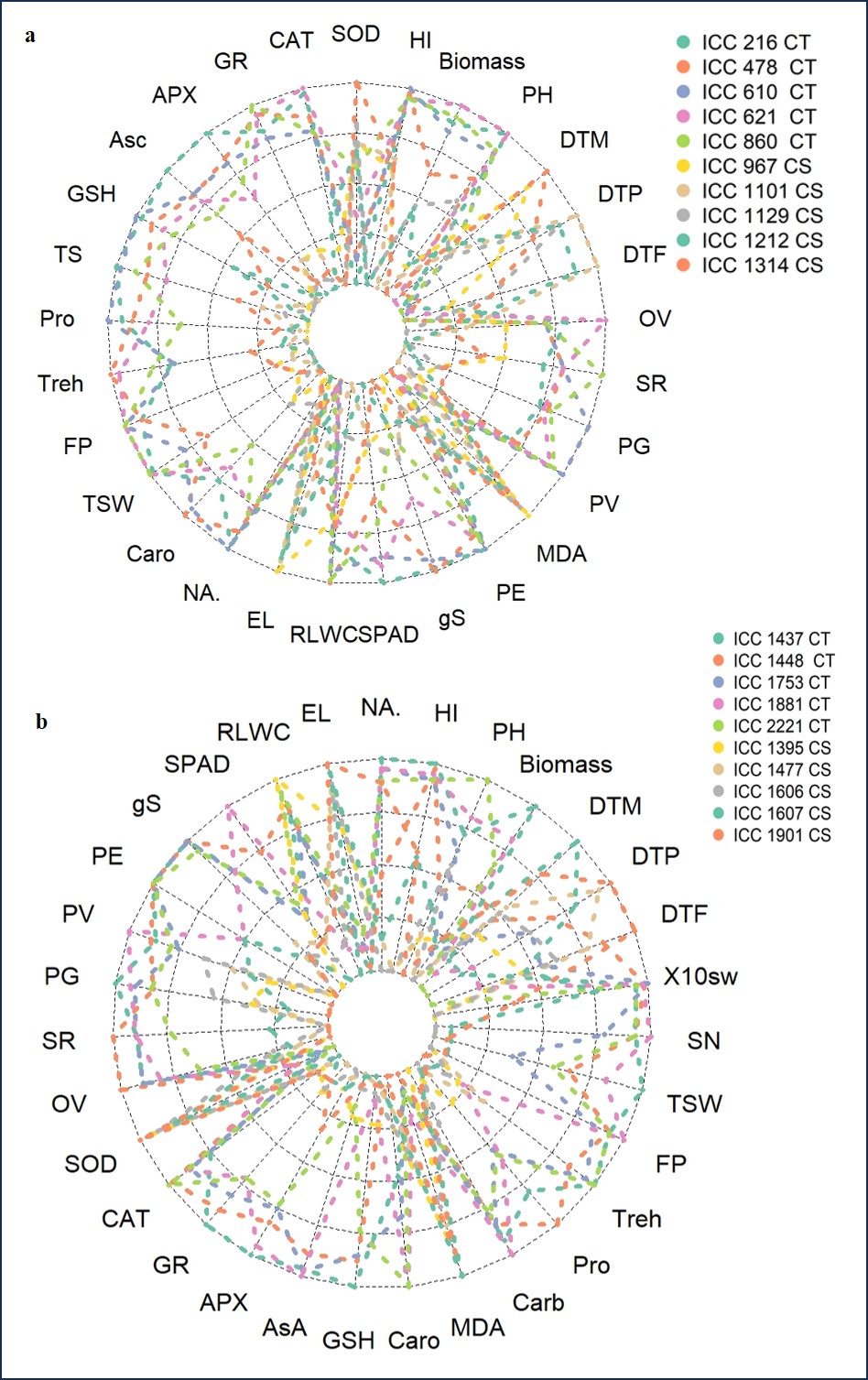


**Figure S11.** Radar plots a) Year 1 and b) Year 2. Normalised mean values of selected genotypes under cold stress.

**Abbreviation:** PH: plant height; DTF: days to flowering, DTP: days to podding, DTM: days to maturity; HI: harvest index; RLWC: relative leaf water content, gS: stomatal conductance; SPAD: SPAD chlorophyll; Caro: carotenoid; Chl. F: chlorophyll fluorescence; NA: nodulation ability; EL: electrolyte leakage; MDA: malondialdehyde content; SOD: superoxide dismutase; CAT: catalase; APX: ascorbate peroxidase; GR: glutathione reductase; AsA: ascorbic acid; GSH: reduced glutathione; PV: pollen viability; PG: pollen germination, SR: stigma receptivity; OV: ovule viability; Pro: proline; TS; total sugars; Treh: trehalose, FP: filled pods; 10-sw: 10-seed weight; TSW: total seed weight; SN: seed number.

Axes: Each radial line represents one trait. The distance from the centre = standardised magnitude (z-score) of that trait. Colour: Different colored points/lines correspond to different CT and CS genotypes. The legend must show which colour = which genotype.
